# Emotional and relational problems of adolescents with and without a migrant background in Europe: a systematic review

**DOI:** 10.1007/s00787-024-02412-y

**Published:** 2024-04-04

**Authors:** Elena Rodríguez-Ventosa Herrera, Isabel Muñoz-San Roque, María Angustias Roldán Franco

**Affiliations:** Comillas Pontifical University, Madrid, Spain

**Keywords:** Adolescent, Migrant, Emotional problems, Mental health, Relational problems, Systematic review

## Abstract

Mental health of migrant adolescents is a topic that has been widely studied in the past decades. Emotional and behavioural problems are amongst the most explored areas; however, little attention has been paid to the relational sphere, which represents another key aspect of mental health and is paramount during adolescence. This systematic review analysed the available evidence on emotional and relational problems comparing adolescents with and without a migrant background in Europe between 2010 and 2021. The search was conducted in four databases using a common search strategy composed of terms addressing adolescence, migrant population, and emotional and relational problems. Three rounds of screening produced 36 eligible studies. Factors affecting both types of problems were identified and categorised using thematic synthesis, dividing them into factors affecting both types of problems jointly or separately and analysing them according to three systemic levels affecting the adolescents’ lives (intrapersonal, interpersonal and external). Critical analysis of the results pointed to mixed findings, with a mild tendency in migrant-background adolescents to portray more emotional problems than their native peers and a stronger tendency for relational problems in the same direction. Several limitations were identified and, along with the conclusions, point to suggestions for future research focusing on studying relational problems as a key component of mental health and its link to emotional problems. Further suggestions entail designing studies that target adolescents with different migrant-background profiles and cultural origins to establish differences between them and identify additional factors affecting emotional and relational problems during the pre-migration and transit phases of the migratory journey to help prevent the onset of these problems.

**Supplementary Information:**

The online version contains supplementary material available at 10.1007/s00787-024-02412-y.

## Introduction

Adolescence represents a period in lifespan which is paramount for the development of the human being. It entails a series of fundamental tasks that must be completed to guarantee a correct development that will set the basis for future adults [1]. Migrant adolescents encounter additional obstacles and burdens related to their specific condition as migrants. Migration has been described as a non-normative critical life event that can entail personal, family, and educational challenges, such as socio-economic problems, family conflicts, lack of peer support, low proficiency in the host language and acculturation stress, among others [2–4]. There are no identical experiences when it comes to the barriers encountered during the pre-migration, transit and post-migration phases, and these are often related to the reasons for migrating and the routes to get to the host country. Disparities have been found for different groups of migrant adolescents, including first-generation and second-generation migrants, economic migrants, unaccompanied migrant minors, asylum seekers and refugees. Mental health assessment of migrant adolescents points to different levels of severity of pathologies depending on the aforementioned migration profiles [5]. This is explained through the exposure to various risk factors and the vulnerability levels intersecting in each case [6, 7]. In fact, some authors consider that being a migrant is a risk factor for developing mental health problems [8, 9]. Several existing systematic reviews on mental health of migrant adolescents have focused on emotional and behavioural problems mostly [10–12], disregarding the relational and social sphere that is central to health, which is supported by the biopsychosocial model and by the self-determination theory of emotional well-being [13]. Social relations, mainly with peers, are a core part of adolescents’ lives and proper development, especially in promoting their self-worth and sense of identity and successfully navigating the integration process and acculturation tasks [14–17]. As a result, relational problems in the social sphere can potentially be detrimental to these adolescents’ correct development and successful integration process, therefore damaging their general well-being and mental health [16, 18]. As there is no universal definition of relational problems, for this study, we describe them as a lack of interpersonal relationships with peers, peer support, friendships, peer acceptance or social competence, or where there are explicit peer relationship problems or peer rejection. Peer acceptance here adopts the definition included in the SSRS questionnaire, which comprises assertiveness, self-control, empathy and cooperation [19]. Bullying and peer victimisation have not been considered as relational problems in this study, nor problems with other relevant people, such as family members or teachers. Although very interesting and relevant, their inclusion is out of the scope of the present study as they entail different domains of social and emotional well-being. For emotional problems, anxiety and depressive symptoms and disorders will be considered since around 4% and 2% of adolescents worldwide develop anxiety and depressive disorders, respectively [20]. Such estimates are not available for the prevalence of relational problems nor for migrant adolescents specifically. The study of these aspects of mental health will enable professionals working with this population in different contexts to obtain a better understanding of their situation and contribute to preventing these problems, which is key to fostering their successful integration in the host country and reducing the mental health gap between migrant and non-migrant adolescents [21]. In the present study we examine whether migrant adolescents present more emotional and relational problems than their non-migrant peers conducting a systematic review. We also aim to identify risk and protective factors included in the selected papers associated with the presence of emotional and relational problems to support the successful prevention of their onset. Based on the additional barriers that migrant adolescents encounter in the different stages of their migratory journey and their potential impact on their well-being, we hypothesize that they will present more emotional and relational problems than their non-migrant peers.

## Method

To guarantee the quality of the systematic review, we designed the method according to the Preferred Reporting Items for Systematic Reviews and Meta-Analyses (PRISMA) [22], and we registered the review in the PROSPERO (International Prospective Register for Systematic Reviews) database of the National Institute for Health Research (NIHR). This database hosts systematic reviews in health and social care, among others, where there is a health-related outcome. It consists of a review protocol that includes the relevant information on the systematic review to be conducted to avoid bias across the process by enabling the comparison of the final review with its initial plan stated in the protocol. We registered the review on the 22nd of April 2022.

### Eligibility criteria

We defined the eligibility criteria following the Preferred Reporting Items for Systematic Reviews and Meta-Analyses [22]. Once we formulated the PICOSS^1^ question, we selected the inclusion criteria to guarantee that the included studies were accurate to provide evidence to answer the research question.

We divided the inclusion criteria into different categories. Regarding publication characteristics, we only included peer-reviewed articles in English, German or Spanish, and the publication period ranged between 2010 and 2021. Although we defined the publication period for the indicated years, we included studies if the data they used were gathered between 2005 and 2021. We established this criterion to ensure that data were still representative of the social moment and not outdated. Regarding the study design, we selected studies operating with quantitative data, and the sample size had to be at least N = 100. However, we made exceptions if the study population was difficult to access (e.g., refugees or asylum seekers) to guarantee that we included these migrant adolescents’ profiles. Additionally, studies had to compare adolescents aged between 11 and 18 with and without a migrant background based in European countries. We considered an adolescent to have a migrant background if a) national frontiers were crossed (1st generation migrant) or b) if one or both parents were foreign-born (2nd generation). We did not consider the third generation in this study. Outcomes had to provide direct measures of the prevalence of emotional or relational problems and be statistically significant.

### Search strategy

The search strategy also followed the PRISMA methodology. The databases consulted were the ones that best fit the research topic, namely, PsycInfo, Psychology and Behavioral Sciences Collection, PubMed, and Web of Science. Although we defined the search strategy between September and December 2021 (including several test searches and refinement of the search terms to be included and excluded), we conducted the final search on the 5th of January 2022. We developed two general search strategies for the definition of the final search strategy and the selection of the search terms. One focused on emotional problems, and the other on relational problems; however, both coincided in the terms included to define the study population referring to adolescent age and migrant background. Once we defined both general strategies, we enriched them with documentary language specific to each database. The resulting search strategies for each database can be consulted in Appendix A for emotional problems and in Appendix B for relational problems. Search areas included title and abstract.

### Study selection

**Fig. 1:**
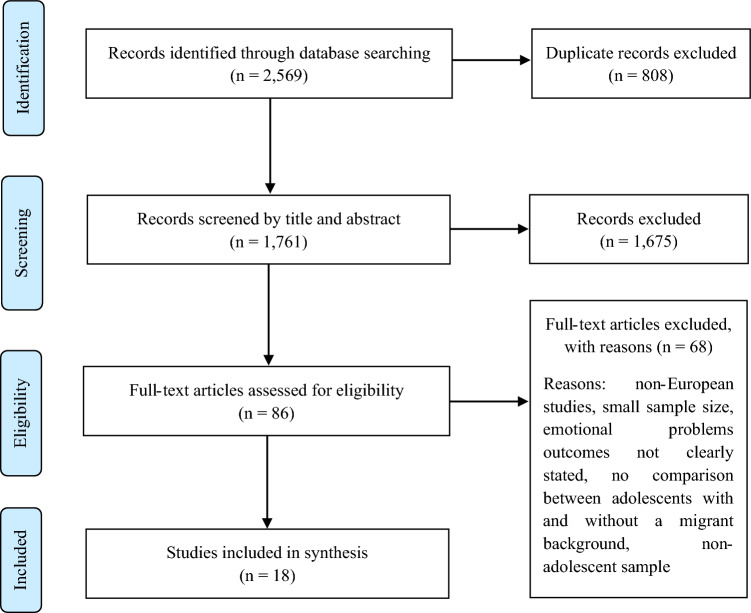
PRISMA flow diagram of studies addressing emotional problems

After applying the designed search strategies in each of the selected databases, we identified a total of 2569 articles for emotional problems. These were incorporated into the Rayyan software for systematic reviews [23], which helped identify the 808 duplicate records that we eliminated, and we also used the software for the following stages of the selection process. The screening process started with 1761 studies, which, after the title and abstract screening based on eligibility criteria, resulted in the exclusion of 1675 studies. We assessed the remaining 86 studies for eligibility based on the full-text screening. This process was carried out independently by the three authors of the present paper, who provided their opinions on the inclusion or exclusion of the remaining articles in the final selection. We compared our three views, and whenever there were disagreements on the final decision on a particular paper, we held a discussion until we made a final joint decision for inclusion. A total of 68 studies were excluded due to not being European, having a small sample size, emotional problems’ outcomes were not clearly stated, there was no comparison between adolescents with and without a migrant background, or the sample not including adolescents. We included the resulting 18 studies in the synthesis. Figure 1 shows the study selection process for emotional problems, which followed the structure of the PRISMA Diagram [22].

**Fig. 2:**
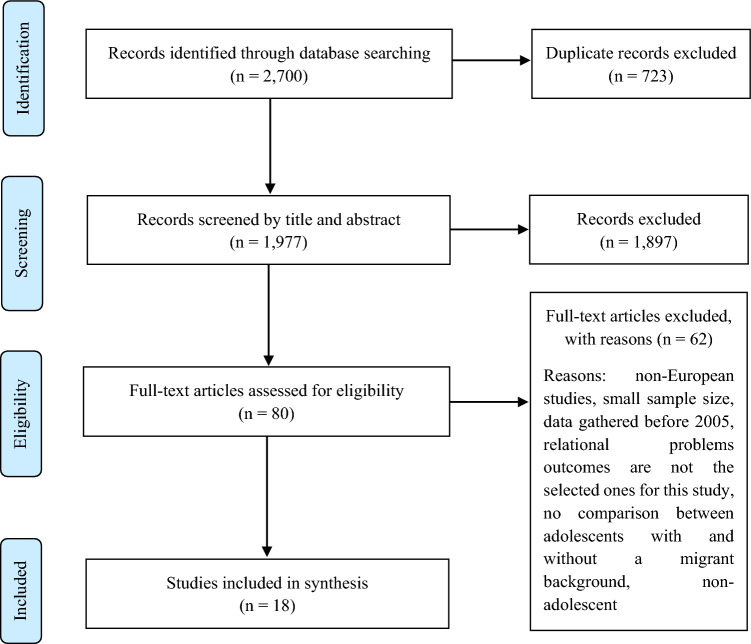
PRISMA flow diagram of studies addressing relational problems

We followed the same procedure for the selection of articles addressing relational problems. The search in the different databases delivered a total of 2700 articles, which resulted in 1977 records undergoing title and abstract screening after the exclusion of duplicate records. The records excluded after this phase were 1897, leaving 80 records for full-text screening to assess them for eligibility. We excluded 62 articles owing to not being European, having a small sample size, being based on data gathered before 2005, the relational problem outcomes did not match the ones selected for this study, there was no comparison between adolescents with and without a migrant background or the sample not including adolescents. Surprisingly, we finally included 18 studies in the synthesis, the same amount as in the search for emotional problems. Figure 2 shows the study selection process on relational problems, which followed the structure of the PRISMA Diagram [22].

### Risk of bias assessment

The selected studies underwent a risk of bias assessment to guarantee they met the required quality criteria. We considered several tools for the process based on the study design, which referred to cross-sectional descriptive studies. Of the tools that assessed these kinds of studies, we chose the JBI (Joanna Briggs Institute) Descriptive Studies Appraisal Tool [24]. According to a systematic review analysing several risk of bias assessment tools by Ma et al. [25], it was one of the most used, the newest among all, and it only included nine items, which were enough to conduct the quality assessment but were not too many in comparison to other tools. We adapted some items to the specificity of the population studied, which, due to difficulty in access, required a smaller sample (item 3, sample size), and the application of tests used self-reports in some cases, which could compromise validity (item 6, use of valid methods for the identification of the condition). However, to safeguard validity, we exclusively included studies using tools that had undergone analysis of their psychometric characteristics and their suitability to be used with the study population or at least with the general adolescent population. Although not all the 18 selected studies on emotional problems and the other 18 on relational problems met the nine items successfully, we identified no risk of bias among those that did not meet some of the items or in which it was unclear. The JBI matrix for risk of bias assessment in cross-sectional descriptive studies for the selected studies addressing emotional and relational problems can be found in Appendix C and Appendix D.

### Data abstraction

We extracted data from articles on emotional and relational problems considering different key aspects included in the selected studies. First, we extracted key characteristics of the studies and organised them into two tables (Appendix E and Appendix F), where data were reported on the migrant profile, sample size, whether it was an institutionalised sample, age, country of destination, country of origin, whether the study compared the outcomes by origin, the definition of migrant and native adolescent provided in the study (to provide evidence of the lack of coherence in the definitions of migrant background among studies), the emotional or relational problems’ outcomes considered in the study, the measures used to evaluate the prevalence of the problem, and the problem behaviour report (whether it was the adolescents themselves or their parents/teachers who answered the questionnaires). Second, in line with the hypothesis held in the present study, we paid more specific attention to the outcomes, identifying whether the studies found statistically significant differences in the emotional and relational problems explored between both adolescent groups. Finally, we also extracted data on the relevant socio-demographic or situational characteristics that affected the outcomes positively or negatively.

### Acceptance or rejection of the hypothesis

In order for the hypothesis to be accepted in the present study, at least 75% of the included studies on emotional or relational problems must provide evidence that adolescents with a migrant background present more emotional and/or relational problems than their non-migrant peers. The decision to set this threshold was based on the criterion used in Delphi methodology, which seeks to find consensus among experts in the field. In a systematic review conducted on Dephi studies, it was found that 39,9% of the included studies defined consensus as 60% agreement or higher, where 75% was the median value [68]. We opted to include the median value as our definition for consensus since it was stricter. We considered it needed to be this way since the acceptance or the rejection of the hypothesis in this case directly depended on the selected studies.

## Results

In light of the information extracted from the included articles, we have divided the results into different key aspects that include the characteristics of the migrant population in the selected studies, the differences found by migrant background in emotional and relational problems, and the factors affecting the latter.

### Characteristics of the migrant population in the included studies

#### Definition of ‘migrant’

There is no unified definition of ‘migrant’, not even of first- and second-generation migrant. Therefore, it is not surprising that there is no consensual definition of a native person in the migration literature either. The studies included in the present paper are no exception in this regard. While five out of 36 studies hold the exact definition of first-generation migrant, second-generation migrant and native [3, 16, 26–28], the rest of the studies either hold slightly different definitions of these terms or do not define what they consider to be a migrant or a native.

Some studies only provide their generic definition of migrant without differentiating between generations. For instance, two studies focus on nationality, defining those who have a different nationality from the host country as migrants [29, 30]. Another two studies focus on the parents’ origin, meaning that either one or both parents should be born abroad for an adolescent to be considered a migrant [31, 32]. As they do not point to where the adolescent was born, this definition could include first- and second-generation migrants according to our definition. Finally, another two studies consider that an adolescent can be defined as a migrant if they and both parents were born abroad, which is what we consider a first-generation migrant, according to our definition [33, 34].

Regarding the definitions of first-generation migrants, all included studies that provide a definition align with ours [3, 16, 26–28, 35–40]. However, three of them specifically mention that the adolescent and at least one parent should be foreign-born [41–43]. While we automatically consider that an adolescent is a first-generation migrant if he or she was born abroad, these three studies also add the condition that at least one parent should be born abroad as well. Technically, the adolescents included in this category would all be the same, but these three articles go a step further and provide specific information regarding both parents’ origins.

Second-generation migrants are also defined by almost all included studies which evaluate their results for this profile in the same way as our definition, meaning that the adolescents are native-born and at least one of the parents is born abroad [3, 16, 26, 28, 36, 42, 43]. Nevertheless, five studies add to their definition that both parents have to be born abroad [35, 37–40]. Finally, studies also differ in their definition of native adolescents. Most papers that define a native adolescent coincide with our definition [3, 16, 26–28, 31, 32, 35, 39, 42, 43]. However, four studies considered a child with at least one native parent as a native [36–38, 40], while that would fit our definition of a second-generation migrant. Only one study focuses on nationality to define the participant’s background [29].

While all included studies provide results for migrant adolescents compared to native adolescents, only a few focus on specific migrant profiles. Five studies provide evidence of emotional and relational problems in first- and second-generation migrant adolescents, comparing them to their native peers [4, 35–37, 43]. Two studies compare refugee adolescents to their native peers [33, 34]. Finally, only one study targets unaccompanied minors and compares them to their non-migrant peers [44].

#### Countries of destination and countries of origin of participants with a migrant background

This study focuses on the emotional and relational problems of adolescents with and without a migrant background in Europe. Nevertheless, the selected papers solely represent 11 European countries. Seven studies focus on Italy [27, 37–39, 42, 44, 45], five in the Netherlands [28, 31, 32, 45, 46], four in Germany [32, 47–49] and Spain [29, 50–52], three in Austria [3, 36, 53], Sweden [16, 35, 40] and Turkey [33, 34, 54], two in Greece [4, 55], Portugal [51, 52] and Switzerland [30, 56] and only one in Norway [26] and in several European countries [43].

Surprisingly, of the 36 included studies, seven provide specific information about the countries of origin of the migrant-background adolescents, enabling comparisons by the origin of the participants. Dutch adolescents are the population that got the most compared by the selected studies, including non-Western adolescents [31], Moroccan adolescents [28], Surinamese, Turkish and Moroccan Dutch adolescents [45, 46]. Germans are compared to Asians [47] and other European adolescents [48]. Italian adolescents are compared to Eastern European, non-Western non-European and Western European migrant adolescents [42, 57]. Finally, Spaniards are compared to Latin-American migrant adolescents [50].

### Differences found by migrant background in emotional problems

To verify if the included studies found results in line with our hypothesis, we analysed whether they found clear evidence that adolescents with a migrant background portrayed more emotional problems than their native peers, fewer problems or no significant differences between both groups. Emotional problems mainly refer to depression, anxiety and related emotional symptoms, including suicidal thoughts. In this analysis, we also highlighted the specific origin of the migrant-background adolescents if this sample was composed of at least 100 adolescents, enabling significant comparisons and extracting conclusions. As many studies did not describe the background of the migrant adolescents in their sample or this group was too small, we provide this information in a few cases.

A total of 10 papers found results that aligned with the hypothesis, as portrayed in Table 1. Seven of them measured the differences between the two groups in anxiety problems and symptomatology [33, 36, 44, 47, 49, 53, 54]. Only one of them included refugee adolescents [33], another one included unaccompanied migrant children [44], and another one measured social anxiety and differentiated it between first and second generation, finding that the first generation showed more anxiety symptoms, followed by the second-generation adolescents and finally by adolescents without a migrant background [36]. Nine papers which concentrated on depression and depressive symptomatology found results that coincided with the hypothesis, providing evidence that having a migrant status was associated with higher depression scores [3, 30, 33, 36, 44, 47, 49, 50, 53]. Again, Karadag and Ogutlu [33] were the ones including refugee adolescents compared to adolescents without a migrant background and Thommessen et al. compared unaccompanied migrant minors to their native peers [44]. This last study included the social workers’ and parents’ views of the adolescents rather than their own, which could produce a bias in the results found. Strohmeier and Dogan [36] found that first-generation migrant adolescents presented higher depression levels than their second-generation peers, and these were higher than their peers without a migrant background. Regarding the origins of the migrant-background adolescents, Turkish adolescents seemed to portray higher depression and anxiety scores than their Austrian native peers [36, 53], and Asian migrant adolescents had higher scores than their German peers [47]. However, although the Asian sample was small (n = 71), the differences between both groups were still statistically significant.

**Table 1:** Alignment of the results on emotional problems with the central hypothesis

Study	Opposite to the hypothesis	No differences between groups	In line with the hypothesis
[29]		Depressive and anxious symptomatology M/N	
[48]	Depression European M < N	Depression Other origins M/N	
[47]		Depressive and anxious symptomatology Other origins M/N	Depressive and anxious symptomatology Asian M > N
[26]		Depressive symptomatology M/N	
[49]			Depressive symptomatology M > N Anxiety symptomatology M > N
[30]		Anxiety M/N	Depression M > N
[53]			Depression Turkish M > N State Anxiety Turkish M > N
[56]	Suicidal tendencies M < N	Depression and anxiety M/N	
[33]			Depressive and anxious symptomatology MR > N
[27]		Depression M/N	
[28]	Depression Moroccan M < N	Generalised and social anxiety Moroccan M/N	
[50]	General Anxiety Latin-American M < N		Depressive symptoms Latin-American M > N
[3]			Depressive symptoms M1 > M2 > N
[36]		Depression Turkish M2/N Social Anxiety Turkish M2/N	Depression Turkish M1 > M2&N Social anxiety Turkish M1 > M2&N
[44]			Depressive and anxious symptomatology UAMsw > Np
[54]			Social anxiety M > N
[46]	Depression and anxiety Moroccan M < N T1&T2 Surinamese & Turkish M < N T2	Depression and anxiety Moroccan M/N T1 Surinamese & Turkish M/N T1	
[45]	Depression and anxiety Moroccan Mp < Np	Depression and anxiety Surinamese & Turkish Mp/Np M/N	

M > N (migrants more problems than natives); M/N (studies were inconclusive, differences were found without statistical significance, or results were similar between both groups); M < N (migrants fewer problems than natives); M = migrant; N = native; M1 = first-generation migrant; M2 = second-generation migrant; Mp = migrant parent; Np = native parent; MR = migrant refugee; UAMsw = social worker of an unaccompanied migrant minor; T1 = first time when the results were measured; T2 = second time when the results were measured (T1 and T2 refer to longitudinal studies)

The remaining papers that did not align with the central hypothesis showed two different results; they either found that migrant adolescents presented fewer emotional problems than their peers born in the host country or they found no significant differences between both groups. A total of six papers found more emotional problems in native adolescents than in their migrant-background peers [28, 45, 46, 48, 50, 56]. Four studies focused on depression, with one of them finding that European adolescents with a migrant background had lower levels than their German peers [48] and the remaining finding the same results when comparing Moroccan adolescents to their native Dutch peers [25, 45, 46] or Surinamese and Turkish adolescents compared to their Dutch peers [46]. Three studies focused on anxiety [45, 46, 50], one finding higher anxiety levels in Spanish adolescents than in their Latin-American peers [50], two of them finding higher anxiety levels in Moroccan adolescents than in their native Dutch peers [45, 46] and one comparing Surinamese and Turkish adolescents to Dutch adolescents [46]. Interestingly, one of these studies found this result exclusively for parental perceptions of depression and anxiety levels of their offspring, while this was not shared by the adolescents [45]. Additionally, one study found more suicidal tendencies in Swiss adolescents than in their peers with a migrant background [56].

Among the eleven articles that found no statistically significant differences between both groups, nine focused on depression or depressive symptomatology [26, 27, 29, 36, 45–48, 56] and eight on anxiety disorders or symptomatology [28–30, 36, 45–47, 56]. Regarding the studies that provided information on the migrant background of the adolescents, one found no differences in emotional problems between adolescents originating from countries different from some European countries, including Turkish, Polish, Russian, Arabic, African, Kurdish and Asian and their German peers [48], and another one between Turkish, Kurdish, Russian, other European countries, Polish, Arabic and African adolescents and their German peers [47]. Two studies found no significant differences between Moroccan adolescents and their Dutch peers [28, 46], two studies between Surinamese and Turkish adolescents and their Dutch peers [45, 46], and one study between Turkish adolescents and their Austrian peers [36]. Only one study found no significant differences between second-generation migrant adolescents and their native peers [36].

### Differences found by migrant background in relational problems

To analyse whether the included studies found results that aligned with the hypothesis, we looked into the evidence that either found more relational problems in migrant-background adolescents than in their non-migrant peers, no statistically significant differences between both groups or more problems in natives than in their migrant-background peers. In this analysis, we also highlight the migrant-background adolescents’ specific origin if this sample comprises at least 100 adolescents. As many studies did not describe the background of the migrant adolescents in their sample or this group was too small, we provide this information in a few cases. Table 2 shows the comparisons of relational problems between adolescents with and without a migrant background and their alignment with the hypothesis in all included studies.

**Table 2:** Alignment of the results on relational problems with the central hypothesis

Study	Opposite to the hypothesis	No differences between groups	In line with the hypothesis
[37]			Peer friendship M1 < M2 < N Peer acceptance M < N
[55]	Peer acceptance M > N	Peer rejection T2 M/N	Peer rejection T1 M > N Peer acceptance T1 & T2 M < N
[38]		Peer acceptance M/N	Peer friendship M < N
[57]		Peer support Western M/N	Peer support EE/nW-nE M < N
[39]		Peer acceptance M/N Peer popularity M/N	
[42]		Peer support Western M/N	Peer support EE/nW-nE M < N
[43]			Peer support M1 < M2 < N
[31]			Peer relationship problems nW M > N
[16]			Peer relations Asian, African and other origins M1 < N
[34]			Peer problems MR < N
[33]			Peer problems MR < N
[4]		Peer popularity T2 M/N	Peer popularity T1 M1 < M2 < N
[51]		Peer acceptance M/N Spanish	Peer acceptance M < N Portuguese
[40]			Peer rejection M1 > M2 > N Peer friendship M < N
[52]		Social competence M/N	Peer acceptance M < N Peer friendship M < N
[32]			Peer friendship M < N
[35]	Peer friendship M2 > N		
[54]			Peer relations M > N

M > N (migrants more problems, acceptance, popularity, friendships or social acceptance than natives); M/N (studies were inconclusive, differences were found but without statistical significance); M = N (same results in both groups); M < N (migrants fewer problems, acceptance, popularity, friendships or social acceptance than natives); M = migrant; N = native; M1 = first-generation migrant; M2 = second-generation migrant; MR = migrant refugees; EE M = Eastern European migrant; nW M = non-Western migrant; nE M = non-European migrant; T1 = first time when the results were measured; T2 = second time when the results were measured (T1 and T2 refer to longitudinal studies)

A total of 16 papers aligned with the central hypothesis regarding relational problems [4, 16, 31–34, 37, 38, 40, 42, 43, 51, 52, 54, 55, 57]. Seven papers focused on peer friendship and peer relations [16, 32, 37, 38, 40, 52, 54], one of them finding that first-generation migrant adolescents had a lower number of friends than second-generation migrant adolescents and their native peers [37]. Regarding the origin of the migrant-background adolescents, only one study provided this information, finding that first-generation Asian and African adolescents had fewer peer relations than their Swedish peers; they came across the same finding for first-generation migrant adolescents from other unspecified origins [16]. Five studies analysed the differences in peer acceptance and popularity, finding that migrant adolescents were less accepted by their classmates and less popular than their non-migrant peers [4, 37, 51, 52, 55]. One longitudinal study that provided results for the different migrant-background generations found that the first time it was measured, first-generation migrant adolescents were less popular than second-generation and non-migrant adolescents [4]. Another longitudinal study found that peer acceptance was higher in native adolescents, measured at two different moments [55]. One study comparing peer acceptance levels between migrant-background adolescents and their Spanish and Portuguese native peers found better results for the non-migrant Portuguese adolescents [51]. Opposite to peer acceptance, two studies measured peer rejection, finding that migrant-background adolescents were more rejected than their native peers, and one of them also provided evidence that first-generation migrant adolescents were more rejected than second-generation migrants and their native peers [40, 55]. Three studies focused on peer support [42, 43, 57]. Two found that migrant adolescents from Eastern-European and non-Western non-European countries perceived less peer support than their native Italian peers [42, 57]. The remaining study found that first-generation migrant peers perceived less peer support than second-generation and native adolescents [43]. Three papers measured peer problems [31, 33, 34]. Two found that refugee adolescents had more problems than their non-migrant peers [33, 34], and another one found that migrant adolescents from non-Western countries accounted for more peer relationship problems than their Dutch peers [31].

Eight studies found no significant differences in relational problems when comparing adolescents with and without a migrant background. Four focused on peer acceptance and popularity [4, 38, 39, 51], two on peer support [42, 57], one on peer rejection [55] and one on social competence [52]. Regarding their origins, the articles on peer support found no differences between migrant-background adolescents from Western countries (including EU-15 and other Western countries such as Switzerland, Norway, Iceland, the USA, Canada, Australia and New Zealand) and their Italian peers [42, 57]. Another study, which compared peer acceptance of migrant-background adolescents and their non-migrant Spanish or Portuguese peers, found no differences between groups only when compared to Spanish native peers [51]. The study that focused on peer rejection was a longitudinal study that found no differences between groups the second time it was measured [55]. Two studies found results opposite to the hypothesis, one of them accounting for more peer acceptance in migrant-background adolescents than in their native peers [55] and another one finding that second-generation migrant adolescents had more peer friendships than their non-migrant peers [35].

### Factors affecting emotional and relational problems

We used thematic synthesis to extract the variables affecting the development of emotional and relational problems [59]. This process consists of three phases, which entail 1) the identification of factors influencing the development of emotional and/or relational problems, 2) the development of descriptive themes that summarise the meanings of the initial variables identified and 3) the generation of analytical themes that enable the interpretation of the previously identified descriptive themes to generate new synthetic categories that enable answering our research question.

The thematic synthesis delivered six descriptive themes (socio-demographic, psychological, family factors, abnormal environment, immigrant proportion of the classroom and time) and three analytical themes (variables affecting emotional problems, relational problems or both emotional and relational problems). We developed the descriptive themes to group the identified variables in the papers included in this review, while analytical themes enabled to interpret the descriptive themes concerning the key outcome, which is the presence of emotional and relational problems. To explain the results, we conducted the analysis according to the three analytical themes, and within each of them, we also contextualised the descriptive themes in the different systemic levels (intrapersonal, interpersonal and external). The factors affecting mental health identified in the study can be found in Table 3.

**Table 3:** Identified factors affecting emotional and relational problems in three systemic levels

	Identified variables affecting emotional and relational problems	
Systemic levels	Descriptive themes	Subthemes
Intrapersonal	Socio-demographic	Age Gender Ethnicity
	Psychological	Self-concept *Cognitive ability* IQ
Interpersonal	Family factors	Structure*
		Relations
		*Culture and attitudes*
		SES
		Situational*
External	Abnormal environment	
	*Immigrant proportion of the classroom*	
	*Time*	

Regular font stands for factors found in included papers addressing both emotional and relational problems; underlined font for factors found in papers addressing emotional problems; and *italics* for factors found in papers addressing relational problems, coinciding with the analytical themes. *Family structure refers to the configuration of the family (e.g., single-parent family vs family with both parents). Situational family factors refer to losing a relative (e.g., mother, father or sibling) or living with a family member with a psychopathology that affects the family dynamic (usually in a negative way)

#### Factors affecting both emotional and relational problems

The first analytical theme includes factors found in the studies that affected both emotional and relational problems. At the intrapersonal level, socio-demographic characteristics were the first descriptive theme found to affect both emotional and relational problems. We found gender differences in emotional and relational problems in 13 papers [3, 27, 30, 37, 38, 40, 42, 45, 47, 48, 50, 51, 53]. Girls seemed to be more prone to developing emotional problems than boys in seven studies [3, 27, 30, 45, 47, 48, 50]; however, one study found that boys had higher anxiety levels than girls [53]. Six additional studies found no gender differences in emotional problems, specifically referring to emotional symptoms [29, 36], depression [28, 33, 53], suicidal tendencies [56], and anxiety [28, 33]. For relational problems, similar results were found, with four studies accounting for worse results in girls than in boys regarding peer friendship, acceptance and support [37, 38, 42, 51] and only one finding more peer rejection in boys [40]. Six papers found no gender differences in relational problems regarding peer acceptance [38, 39, 52], popularity [4, 39], friendship [52], and peer relationship problems [31, 33].

Five papers also found ethnicity to affect emotional and relational problems [42, 45, 47, 48, 57], providing mixed findings. On the one hand, regarding the emotional problems of German native adolescents, two studies provided different results depending on who they were compared with. Asians reported more internalising problems than their German peers. Still, several authors did not find this result for adolescents with a different migrant background, and they found the opposite effect when compared with their peers with migrant backgrounds from other European countries [47, 48]. Another study found no ethnic differences in emotional problems reported by adolescents; however, they did when asking the parents, where Moroccan Dutch parents reported fewer anxiety disorders than Surinamese Dutch, Turkish Dutch and native Dutch parents [45]. Regarding relational problems, two studies found that adolescents from Eastern Europe and non-Western and non-European countries were likelier to perceive low peer support than their peers from the host country (Italy) or Western countries [42, 57].

At the interpersonal level, some family factors also appeared to influence both emotional and relational problems. Socio-economic status (SES) seemed to affect both types of problems. The included studies held many different definitions of this variable, some focusing on social class [29], parental education [16, 29, 30, 33, 45–48], parental education and household income [40], parental education and occupation [27, 32, 36, 49], socio-economic adversity [4], access to a private room at home [30], OECD definition of SES [37, 52, 60], and SES According to the Family Affluence Scale (FAS) [31, 35, 42, 43, 57, 61]. In most cases, these were self-reported by the adolescents or their parents, except for the household income, which was drawn from the registers held by Statistics Sweden [40]. Some papers described the SES differences between the migrant and non-migrant adolescent groups they were comparing. Out of the 13 papers describing them, 12 found that the migrant adolescents had lower SES than their native peers [27, 29, 31, 33, 36, 42, 43, 46, 49, 52, 54, 57], and just one adjusted for SES in their analyses because they could not match the groups on socio-economic status as the differences between groups were too large [28]. On the one hand, regarding SES effects on emotional problems, two studies found that having a higher SES worked as a protective factor [30, 50], one found that parents’ middle educational background acted as a risk factor [49], and another one found no effects [29]. On the other hand, regarding SES effects on relational problems, having a higher SES was related to more friendships and peer acceptance [37] and fewer peer relationship problems and rejection [31, 40]. However, when controlling for migrant status, it was not a risk factor for popularity [4]. Situational family factors like losing a family member or living with psychopathology predicted the development of depression, anxiety disorders and peer problems [33, 48].

#### Factors affecting emotional problems

The second analytical theme refers to factors found in the articles that affected emotional problems exclusively. Of all included studies, age seemed to be the only socio-demographic descriptive theme at the intrapersonal level affecting depression, where older adolescents had higher levels than their younger peers [26, 27]. Although more studies included age as a variable in their designs, they either found no significant differences or controlled for this variable in their analyses. Additionally, in papers where the study sample included children younger than 11, our study did not include significant age differences as we focused on adolescents aged 11 to 18. Regarding psychological characteristics, having a negative self-concept [49] and average or above-average intelligence levels [47] were associated with increased emotional problems. Family factors at the interpersonal level, such as family structure and family relations**,** seemed to affect the development of emotional problems. Adolescents living in a single-parent family were more prone to developing internalising disorders than children living with both parents [47]. Additionally, perceived maternal and paternal care acted as a protective factor against depressive symptoms [27], while inadequate parental authority or unreasonable demands of parents worked as a risk factor [47]. At the external level, living in an abnormal environment (such as an institution or a household with very limited space) increased the probability of developing anxiety and depressive disorders [47].

#### Factors affecting relational problems

The final analytical theme includes factors that affect relational problems. At the intrapersonal level, within the descriptive psychological theme, having stronger cognitive skills appeared to positively impact rejection, reducing its likelihood [40]. Among the family factors belonging to the interpersonal level, culture and attitudes seemed to play an important role in the development of relational problems. Parents with out-group friends seemed to help migrant children develop positive out-group attitudes that contributed to increasing the number of out-group friends. However, the study did not find the same result for children without a migrant background [32]. At the external level, the immigrant proportion of the classroom seemed crucial in developing relational problems. Four studies found that classrooms with a lower percentage of migrant students had protective effects against the development of relational problems, meaning that they would more likely have more friends without a migrant background, peer acceptance and popularity [4, 32, 35, 55]. Two papers found opposite results, finding that a higher immigrant proportion in the classroom was related to more peer acceptance and less peer rejection [40, 55]. One study found that a lower immigrant proportion in the classroom was associated with higher peer rejection [40]. Time was another descriptive theme identified to affect the development of relational problems. Two longitudinal studies found that over time, adolescents with a migrant background became less rejected, more accepted and more popular than at the first time these were assessed [4, 55].

## Discussion

In the present study, we conducted a systematic review following the PRISMA statement [22], focusing on two main components of the mental health of adolescents in Europe with and without a migrant background: emotional and relational problems. We consulted a vast number of articles to make the final selection for the study. However, we only included 36 studies which met the inclusion criteria. In this sense, one of the most recent systematic reviews evaluating the mental health of migrant children more than a decade ago mentioned that research on this topic was very scarce and urged researchers in the field to keep focusing and deepening on it [10]. After conducting this study, it becomes evident that the topic has received more attention in recent years and continues to be relevant at present.

According to the central hypothesis, adolescents with a migrant background would present more emotional and relational problems than their peers without a migrant background. The discard of many of the consulted papers in the selection process poses a reason to argue that there could be a bias in the results due to the inclusion criteria. Although the studies included portray mixed findings, 26 of the 36 included studies found results in line with our hypothesis for both emotional and relational problems. This study also enabled us to identify factors that influence these problems, pointing to some affecting both emotional and relational problems and others being specific to each type of problem.

Despite having analysed emotional and relational problems separately in this study, both represent key components of well-being. Although the emotional sphere is central to mental health, relatedness to others is considered a peripheral aspect contributing to well-being [62]. According to the structural model of child well-being, emotional and social dimensions are two central components [63]. The emotional dimension holds strong bi-directional connections to all other components, including the social sphere. Therefore, a positive balance in emotional well-being should have a positive effect on the social dimension, and the same would happen if there was a positive or negative balance in the social dimension, meaning that it would either positively or negatively affect the emotional dimension. This conception points to the importance of considering both when researching children’s and adolescents’ mental health and well-being.

As previously stated, emotional problems have been widely studied as a key component of mental health, and the findings of the present study are in line with other systematic reviews evaluating concrete disorders such as depression and anxiety [10, 11, 64]. Although the question regarding whether children with a migrant background present more emotional problems than their peers without a migrant background cannot be fully answered due to several factors that are explained in the following paragraphs, there is a mild tendency among the included studies to consider that they do present more emotional problems, providing evidence that endorses the hypothesis in 10 out of 18 studies. However, based on the established criterion for accepting the hypothesis, it cannot be accepted for emotional problems since less than 75% of the included studies found more emotional problems in migrant adolescents than in their non-migrant peers.

Regarding relational problems, in this study, we provided a definition for these as part of mental health, focusing mainly on peer problems and social competence. We consider these the social part of mental health, usually set aside. However, according to the bio-psycho-social model of health, social factors are as essential as biological factors (such as predisposition to develop certain mental health diseases) and psychological factors, which are the most widely known in the domain of mental health problems. From a developmental psychology perspective, the social sphere is also crucial in the developmental stage of the population studied. In adolescence, the peer group is a key aspect which influences well-being to a large extent. Hence, when researching the mental health of migrant adolescents, it is paramount to focus on their peers and their social competence, which is needed to establish positive peer relationships. In light of the hypothesis stating that adolescents with a migrant background portray more relational problems than their non-migrant peers, there is also a difficulty in providing a final answer due to the same reasons previously mentioned. Nevertheless, the tendency of the included studies points to the hypothesis being true more clearly than in emotional problems, with 16 out of 18 studies in line with it. Therefore, based on our pre-established criterion, the hypothesis is true for relational problems, since more than 75% of the included studies support it. An interesting finding of the present paper is that out of the 18 selected studies on relational problems, only one focused on social competence [52]. Therefore, we suggest that researchers focusing on relational problems specifically dedicate some efforts to evaluating adolescents’ social competence with and without a migrant background to provide more evidence for this underrepresented component of the relational sphere.

Although a majority of studies evaluating emotional and relational problems found results in line with the hypothesis, another significant amount either found no differences between both groups of adolescents or identified more problems in native adolescents than in their migrant peers. To better understand the differences in these findings, we provide more details of the studies that might explain at least part of them. Many identified reasons that could account for bias in the results are related to the study samples. In many studies, the native sample was over-represented compared to the migrant-background sample [26]. This sampling strategy is usually justified stating that it is highly representative of the proportions of the population with and without a migrant background in a particular country; however, it can also explain part of the results found against the hypothesis as it is a convenience sample. Along the same line, some papers that found evidence against the hypothesis pointed to using a convenience sample as a limitation in their studies, which can directly affect the results [26]. Other studies focusing directly on the mental health problems of adolescents referred that their results could partially be explained by the oversampling of at-risk adolescents [28, 46].

Other possible reasons for finding results that refute the hypothesis are related to differences in several factors affecting emotional and relational problems in both populations that were either not controlled or not measured. For instance, one study indicated that the personal history of their participants was not considered in data analysis and suggested that their results represented more of a ‘patchwork’ than the clear picture [56]. Additionally, other reasons might be related to the study design. As this review includes a few longitudinal studies, we observed that, especially for relational problems, differences were found between native and migrant adolescents when comparing them at time one and time two [35, 55]. Usually, the evidence found that supported the hypothesis at T1 was no longer the same at T2, pointing to time as a key variable affecting relational problems. Moreover, studies that sampled classrooms with different compositions of migrant-background students found opposite results depending on the proportions [55]. Finally, the finding shared by some studies that second-generation migrant adolescents and their non-migrant peers had similar levels of emotional or relational problems can be explained by the fact that they were raised in the same country and, therefore, did not encounter some obstacles or challenges that come with the migration and integration processes, in comparison to their first-generation migrant peers [36, 46].

To identify the variables that affect emotional and relational problems, the study came across several findings. First, we identified some as factors protecting against the development of emotional and relational problems, and we found others to act as risk factors. Second, we found that some widely studied variables affected emotional and relational problems, but others were specific to emotional problems and others to relational problems. Many variables affecting emotional problems are also well-known, such as age, self-concept, family structure, family relations and an abnormal environment [65–67]. In contrast, the variables affecting relational problems seem specific for adolescents with a migrant background. These variables include culture and attitudes, the immigrant proportion of the classroom, and time. This finding suggests that these variables and relational problems as part of mental health should be further studied to prevent the development of relational problems in adolescents with a migrant background. Third, we found variables affecting both emotional and relational problems at all three ecological levels, including gender, ethnicity, socio-economic status and situational family factors. This fact points to the importance of conducting research and interventions that tackle intrapersonal, interpersonal and external factors, as all three contribute to adolescents’ mental health and are, therefore, equally important. The knowledge of how these variables influence emotional and relational problems enables professionals working with migrant adolescents to get a broader understanding of how their environment’s important relationships and factors affect them. It gives them the opportunity to design strategies to control them and prevent the onset of such problems.

Delving into some of these key variables affecting both emotional and relational problems, we found that the majority of the included studies pointed to girls portraying more problems than their male peers, especially regarding emotional problems. At the same time, this was less clear for relational problems. Drawing conclusions on ethnicity effects is cumbersome since few origins and host countries are represented, and some studies group several countries together. This grouping hinders verifying whether migrant adolescents from concrete countries are more prone to developing emotional or relational problems than adolescents with a different background. Among the studies that provided this information, one found that adolescents of European descent presented fewer emotional problems than their German peers. At the same time, the rest of the countries were either African (Moroccan compared to Dutch peers) or South American (compared to Spanish and Dutch native peers). Papers that found no significant differences in emotional problems between both groups compared Dutch peers to Moroccan, Turkish and Surinamese migrant adolescents, and Austrian adolescents to their Turkish migrant peers. For relational problems, this was only found in migrant-background adolescents from Western countries compared to their Italian peers.

Regarding studies finding evidence aligned with the hypothesis, it was found that Asian adolescents portrayed more emotional and relational problems than their German and Swedish peers, Turkish adolescents more emotional problems than their Austrian peers, and Latin-American adolescents higher depression scores than their Spanish peers. For relational problems, it was found that African adolescents had fewer peer relationships than their Swedish peers, and migrant adolescents from Eastern-European and non-Western non-European countries had less peer support or more peer problems than their Italian peers. It becomes clear that more research is needed where the samples of different migrant backgrounds are representative and differentiated in the analysis to compare them to their migrant peers and between them. The same can be extrapolated to the native sample, where it would be highly beneficial to establish differences in emotional and relational problems between European backgrounds to provide the prevalence of both types of problems in each country. This information would then enable cross-country studies comparing native and migrant-background adolescents. Finally, while there appears to be a tendency for migrant-background adolescents in the included studies to account for lower SES than their native peers, the different definitions and measurement of SES in the studies prevent us from extracting sound conclusions. Nevertheless, we point to the potential of having high SES as a protective factor that should be further studied.

There are several limitations of the included studies that could imply biases in the results presented. On the one hand, although most of the studies included share the same definition of migrant and native adolescent, some present differences that entail considering an adolescent as a native peer without focusing on the second-generation background. Additionally, as few studies provide different results for first- and second-generation migrants, refugees and unaccompanied minors, we cannot draw any conclusions regarding these groups’ mental health. On the other hand, only 11 European countries were included in the study regarding the cultural factors to be accounted for. While some countries were represented in up to seven studies, others appeared in one study or two, being underrepresented. The same happened with the countries of origin of the migrant background adolescents. A minority of the included articles provided information on the cultural origin of the migrant adolescents, hindering the drawing of conclusions regarding different results based on the specific cultural background.

The present study also faced some limitations that should be carefully considered to contribute to future research in the field. First, we designed differentiated and separate search strategies for emotional and relational problems and included mainly cross-sectional studies, which made it impossible to contribute to studying the mutual dependence and possible causal links between both spheres. Although delving into this link was not a goal pursued by the study, exploring it in future research is extremely interesting, especially considering that both are key elements of well-being closely intertwined. Second, the focus of the present paper on peer relationships only when studying relational problems left out other relevant relationships contributing to both social well-being and the relational sphere of mental health. These are also paramount for the adolescents’ development but were out of the adopted scope. Therefore, we urge future researchers interested in the field to include these relationships in their studies. Third, as we only considered the factors affecting emotional and relational problems included in the studies, we did not focus on additional variables that have proven to affect psychological well-being, such as climate in the family, type of socialization, stereotypes, and size and quality of peer networks, to name a few. These should be included in future research to try to draw causal links between the variables and the emotional and relational problems present. Finally, as this study was designed as a systematic review and not a meta-analysis, we did not provide prevalence estimates for emotional and relational problems found in the studies. The design of the study did not allow it, since the studies included do not share similar sampling strategies, tools or outcomes. Future research should focus on providing such estimates since they are not available for relational problems and especially not related to adolescent migrant population.

## Conclusions

Migration is a complex social phenomenon affecting adults, children, and adolescents. For the latter, the barriers encountered during the pre-migration, transit and post-migration stages of the process pose additional challenges that can impact their mental health.

In the present paper, we conducted a systematic review focusing on emotional and relational problems, identifying 18 articles for each focusing on the European context that measured mental health problems through self-administered questionnaires. Although the inclusion criteria included several profiles of migrant adolescents, very few of the included studies focused on refugees and unaccompanied migrant minors. This finding notes the importance of conducting more comparative research on children without a migrant background and refugees or unaccompanied migrant minors on their mental health status.

Although the hypothesis held in this study points to adolescents with a migrant background presenting more emotional and relational problems than their peers without a migrant background, it could only be confirmed for relational problems, since more than 75% of the included studies support it. A small majority of studies also found more emotional problems in adolescents with a migrant background than in their non-migrant peers. However, as the amount of studies supporting this hypothesis was below the acceptance criterion, it could not be fully confirmed. Nevertheless, to provide more direct evidence to test the hypothesis, more studies are needed focusing on the cultural origins of both adolescents with and without a migrant background, cultural differences between the host and home countries, and the different profiles of migrant-background adolescents. Another critical aspect on which future studies should focus is the factors affecting emotional and relational problems. Many of the factors identified in the included studies, and the most known ones, are embedded in the post-migration stage. However, as already pointed out by Chan and colleagues [10], more research is needed focusing on variables pertaining to both the pre-migration and transit stages and the variables affecting both problems jointly and separately. Moreover, further research is needed to understand the relationship between emotional and relational problems and the key role that these factors play in their presence and onset.

Finally, this study focused on studying the emotional and relational problems of adolescents with and without a migrant background to provide evidence of the differences both peer groups present. It also aimed to identify the variables that affect their mental health, intending to contribute to the prevention and intervention of the development of mental health problems. We urge professionals working with migrant adolescents in different domains and contexts to consider the findings of the present study to tackle the risk and protective factors affecting migrant adolescents and foster their well-being and integration in the host country. This task will help build a better future for these adolescents who slowly enter adulthood and represent a vital part of European countries.

## Supplementary Information

Below is the link to the electronic supplementary material.

Supplementary file1 (PDF 338 KB)

## Data Availability

This declaration is “not applicable”.

## Footnotes

1 PICOSS is an acronym for Participants; Intervention; Comparison; Outcome; Study; and Setting. Participants were adolescents aged 11 to 18 years with and without a migrant background. We did not include intervention, as only prevalence was measured. Additionally, we did not provide prevalence estimates in our study since each study used different measures and sampling strategies and they could not be compared. Therefore, we only informed about whether migrant adolescents or their non-migrant peers portrayed more emotional and relational problems, accounting for statistical significance. Comparison referred to adolescents with a migrant background compared to adolescents without a migrant background. We included quantitative studies in a European setting where a migrant background included international migration of either the adolescents or at least one of their parents.
