# Supplementary material for: Emotional and relational problems of adolescents with and without a migrant background in Europe: a systematic review
Source: Eur Child Adolesc Psychiatry. 2024 Apr 4;33(12):4115–31. doi: 10.1007/s00787-024-02412-y (PMC11618147; doi:10.1007/s00787-024-02412-y)
Supplement: Supplementary file 1 — Supplementary file1 (PDF 338 KB) [file 787_2024_2412_MOESM1_ESM.pdf]

APPENDIX A

Detailed search strategy for articles addressing emotional problems

Date	General search terms	Database	Specific search terms of DDB	Final search strategy	Filters	N total
05/01/2022	(("Depression" OR "Depressive disorder" OR "Depressive Disorders" OR "Anxiety" OR "Anxiety Disorders" OR "Affective disorders" OR "Emotional disturbances" OR "Internalizing Disorders") AND ("Migrant" OR "Migration background" OR "immigrant" OR "Immigrant background" OR "Asylum seek*" OR "Refugee" OR "Unaccompanied migrant minors" OR "UAMS") OR ("child*" OR "adolesc*" OR "teenag*"))	PsycINFO	<u>For emotional problems</u>:<br>DE "Emotional Disturbances"<br>DE "Affective Disorders"<br>DE "Anxiety"<br>DE "Anxiety Disorders"<br>DE "Depression (Emotion)"	(("Depression" OR DE "Depression (Emotion)" OR "Depressive disorder" OR "Depressive Disorders" OR "Anxiety" OR DE "Anxiety" OR "Anxiety Disorders" OR DE "Anxiety Disorders" OR "Affective disorders" OR DE "Affective Disorders" OR "Emotional disturbances" OR DE "Emotional Disturbances" OR "Internalizing Disorders") AND ("Migrant" OR "Migration background" OR "immigrant" OR "Immigrant background" OR "Asylum seek*" OR DE "Asylum Seeking" OR "Refugee" OR "Unaccompanied migrant minors" OR "UAMS") AND ("child*" OR "adolesc*" OR "teenag*"))	1. Period: 2010-2021<br>2. Languages:<br>English, German, Spanish<br>/Castilian	1,178
05/01/2022	(("Depression" OR "Depressive disorder" OR "Depressive Disorders" OR "Anxiety" OR "Anxiety Disorders" OR "Affective disorders" OR	Psychology and Behavioral Sciences Collection	<u>For emotional problems</u>:<br>DE "ANXIETY"<br>DE "ANXIETY in adolescence"<br>DE "ANXIETY disorders"	(("Depression" OR DE "DEPRESSION in adolescence" OR "Depressive disorder" OR "Depressive Disorders" OR "Anxiety" OR DE "ANXIETY" OR DE "ANXIETY in adolescence" OR "Anxiety Disorders"	1. Period: 2010-2021<br>2. Languages:	201

	"Emotional disturbances" OR "Internalizing Disorders") AND ("Migrant" OR "Migration background" OR "immigrant" OR "Immigrant background" OR "Asylum seek*" OR "Refugee" OR "Unaccompanied migrant minors" OR "UAMS") OR ("child*" OR "adolesc*" OR "teenag*"))		DE "DEPRESSION in adolescence"<br>DE "AFFECTIVE disorders"<br><br>For migrant background:<br>DE "IMMIGRANTS" DE "UNACCOMPANIED immigrant children"<br>DE "REFUGEES"<br><br>For adolescent age:<br>DE "CHILDREN"<br>DE "TEENAGERS"<br>DE "ADOLESCENCE"	OR DE "ANXIETY disorders" OR "Affective disorders" OR DE "AFFECTIVE disorders" OR "Emotional disturbances" OR "Internalizing Disorders") AND ("Migrant" OR "Migration background" OR "immigrant" OR DE "IMMIGRANTS" OR "Immigrant background" OR "Asylum seek*" OR "Refugee" OR DE "REFUGEES" OR "Unaccompanied migrant minors" OR DE "UNACCOMPANIED immigrant children" OR "UAMS") AND ("child*" OR DE "CHILDREN" OR "adolesc*" OR DE "ADOLESCENCE" OR "teenag*" OR DE "TEENAGERS"))	English, German, Spanish<br>/Castilian	
05/01/2022	(("Depression" OR "Depressive disorder" OR "Depressive Disorders" OR "Anxiety" OR "Anxiety Disorders" OR "Affective disorders" OR "Emotional disturbances" OR "Internalizing Disorders") AND ("Migrant" OR "Migration background" OR "immigrant" OR "Immigrant background" OR "Asylum seek*" OR "Refugee" OR	PubMed	For emotional problems:<br>"Depression"[Mesh] "Depressive Disorder"[Mesh] "Anxiety"[Mesh]<br><br>For migrant background:<br>"Refugees"[Mesh]<br><br>For adolescent age:	(("Depression" OR "Depression"[Mesh] OR "Depressive disorder" OR "Depressive Disorder"[Mesh] OR "Depressive Disorders" OR "Anxiety" OR "Anxiety"[Mesh] OR "Anxiety Disorders" OR "Affective disorders" OR "Emotional disturbances" OR "Internalizing Disorders") AND ("Migrant" OR "Migration background" OR "immigrant" OR "Immigrant background" OR "Asylum seek*" OR "Refugee" OR "Refugees"[Mesh] OR "Unaccompanied migrant minors" OR	1. Period: 2010-2021<br><br>2. Languages:<br>English, German, Spanish<br>/Castilian	1,150

	"Unaccompanied migrant minors" OR "UAMS") OR ("child*" OR "adolesc*" OR "teenag*"))		"Adolescent"[Mesh] "Child"[Mesh]	"UAMS") AND ("child*" OR "Child"[Mesh] OR "adolesc*" OR "Adolescent"[Mesh] OR "teenag*"))		
05/01/2022	(("Depression" OR "Depressive disorder" OR "Depressive Disorders" OR "Anxiety" OR "Anxiety Disorders" OR "Affective disorders" OR "Emotional disturbances" OR "Internalizing Disorders") AND ("Migrant" OR "Migration background" OR "immigrant" OR "Immigrant background" OR "Asylum seek*" OR "Refugee" OR "Unaccompanied migrant minors" OR "UAMS") OR ("child*" OR "adolesc*" OR "teenag*"))	Web of Science	/	TI/TS=(("Depression" OR "Depressive disorder" OR "Depressive Disorders" OR "Anxiety" OR "Anxiety Disorders" OR "Affective disorders" OR "Emotional disturbances" OR "Internalizing Disorders") AND ("Migrant" OR "Migration background" OR "immigrant" OR "Immigrant background" OR "Asylum seek*" OR "Refugee" OR "Unaccompanied migrant minors" OR "UAMS") AND ("child*" OR "adolesc*" OR "teenag*"))	1. Period: 2010-2021<br>2. Languages:<br>English, German, Spanish<br>/Castilian	40
						**2,569**

APPENDIX B

Detailed search strategy for articles addressing relational problems

Date	General search terms	Database	Specific search terms of DDB	Final search strategy	Filters	N total
05/01/2022	("Peer relations" OR "Peer acceptance" OR "Peer" OR "Peer group" OR "Peer communication" OR "Social skills" OR "Social competence" OR "Social support" OR "Friend*") AND ("Migrant" OR "Migration background" OR "immigrant" OR "Immigrant background" OR "Asylum seek*" OR "Refugee" OR "Unaccompanied migrant minors" OR "UAMS") OR ("child*" OR "adolesc*" OR "teenag*"))	PsycINFO	For relational problems:<br>DE "Peer Relations"<br>DE "Social Skills"<br>DE "Friendship”<br>For migrant background:<br>DE "Asylum Seeking"	(("Peer relations" OR DE "Peer Relations" OR "Peer acceptance" OR "Peer" OR "Peer group" OR "Peer communication" OR "Social skills" OR DE "Social Skills" OR "Social competence" OR "Social support" OR "Friend*" OR DE "Friendship") AND ("Migrant" OR "Migration background" OR "immigrant" OR "Immigrant background" OR "Asylum seek*" OR DE "Asylum Seeking" OR "Refugee" OR "Unaccompanied migrant minors" OR "UAMS") AND ("child*" OR "adolesc*" OR "teenag*"))	1. Period: 2010-2021<br>2. Languages:<br>English, German, Spanish<br>/Castilian	1,256
05/01/2022	("Peer relations" OR "Peer acceptance" OR "Peer" OR "Peer group" OR "Peer communication" OR "Social skills" OR "Social competence" OR "Social support" OR "Friend*") AND ("Migrant" OR "Migration background"	Psychology and Behavioral Sciences Collection	For relational problems:<br>DE "PEER relations"<br>DE "FRIENDSHIP"<br>DE "PEERS"<br>DE "SOCIAL skills"	(("Peer relations" OR DE "PEER relations" OR "Peer acceptance" OR "Peer" OR DE "PEERS" OR "Peer group" OR "Peer communication" OR DE "PEER communication" OR "Social skills" OR DE "SOCIAL skills" OR "Social competence" OR "Social support" OR "Friend*" OR DE "FRIENDSHIP") AND	1. Period: 2010-2021<br>2. Languages:<br>English, German, Spanish<br>/Castilian	246

	OR "immigrant" OR "Immigrant background" OR "Asylum seek*" OR "Refugee" OR "Unaccompanied migrant minors" OR "UAMS") OR ("child*" OR "adolesc*" OR "teenag*"))		DE "PEER communication"<br><br><u>For migrant background</u>:<br>DE "IMMIGRANTS"<br>DE "UNACCOMPANIED immigrant children" DE "REFUGEES"<br><br><u>For adolescent age</u>:<br>DE "CHILDREN"<br>DE "TEENAGERS"<br>DE "ADOLESCENCE"	("Migrant" OR "Migration background" OR "immigrant" OR DE "IMMIGRANTS" OR "Immigrant background" OR "Asylum seek*" OR "Refugee" OR DE "REFUGEES" OR "Unaccompanied migrant minors" OR DE "UNACCOMPANIED immigrant children" OR "UAMS") AND ("child*" OR DE "CHILDREN" OR "adolesc*" OR DE "ADOLESCENCE" OR "teenag*" OR DE "TEENAGERS"))		
05/01/2022	("Peer relations" OR "Peer acceptance" OR "Peer" OR "Peer group" OR "Peer communication" OR "Social skills" OR "Social competence" OR "Social support" OR "Friend*") AND ("Migrant" OR "Migration background" OR "immigrant" OR "Immigrant background" OR "Asylum seek*" OR "Refugee" OR "Unaccompanied migrant minors" OR "UAMS") OR ("child*" OR "adolesc*" OR "teenag*"))	PubMed	<u>For relational problems</u>:<br>"Peer Group"[Mesh]<br>"Social Skills"[Mesh] "Friends"[Mesh]<br><br><u>For migrant background</u>:<br>"Refugees"[Mesh]<br><br><u>For adolescent age</u>:<br>"Adolescent"[Mesh] "Child"[Mesh]	(("Peer relations" OR "Peer acceptance" OR "Peer" OR "Peer group" OR "Peer Group"[Mesh] OR "Peer communication" OR "Social skills" OR "Social Skills"[Mesh] OR "Social competence" OR "Social support" OR "Friend*" OR "Friends"[Mesh]) AND ("Migrant" OR "Migration background" OR "immigrant" OR "Immigrant background" OR "Asylum seek*" OR "Refugee" OR "Refugees"[Mesh] OR "Unaccompanied migrant minors" OR "UAMS") AND ("child*" OR "Child"[Mesh] OR "adolesc*" OR "Adolescent"[Mesh] OR "teenag*"))	1. Period: 2010-2021<br><br>2. Languages:<br>English, German, Spanish<br>/Castilian	1,099

05/01/2022	("Peer relations" OR "Peer acceptance" OR "Peer" OR "Peer group" OR "Peer communication" OR "Social skills" OR "Social competence" OR "Social support" OR "Friend*") AND ("Migrant" OR "Migration background" OR "immigrant" OR "Immigrant background" OR "Asylum seek*" OR "Refugee" OR "Unaccompanied migrant minors" OR "UAMS") OR ("child*" OR "adolesc*" OR "teenag*"))	Web of Science	/	TI/TS=(("Peer relations" OR "Peer acceptance" OR "Peer" OR "Peer group" OR "Peer communication" OR "Social skills" OR "Social competence" OR "Social support" OR "Friend*") AND ("Migrant" OR "Migration background" OR "immigrant" OR "Immigrant background" OR "Asylum seek*" OR "Refugee" OR "Unaccompanied migrant minors" OR "UAMS") AND ("child*" OR "adolesc*" OR "teenag*"))	99
					**2,700**

APPENDIX C

Matrix for risk of bias assessment within emotional problems studies using JBI (The Joanna Briggs Institute, 2011)

Reference	1<br><br>Was the sample frame appropriate to address the target population?	2<br><br>Were study participants sampled in an appropriate way?	3<br><br>Was the sample size adequate?	4<br><br>Were the study subjects and the setting described in detail?	5<br><br>Was the data analysis conducted with sufficient coverage of the identified sample?	6<br><br>Were valid methods used for the identification of the condition?	7<br><br>Was the condition measured in a standard, reliable way for all participants?	8<br><br>Was there appropriate statistical analysis?	9<br><br>Was the response rate adequate, and if not, was the low response rate managed appropriately?
Alonso-Fernández et al. (2017)	Yes	Yes	Yes	Yes	Yes	Yes	Yes	Yes	Yes
Belhadj Kouider et al. (2014)	Yes	Yes	Yes	Yes	Yes	Yes	Yes	Yes	Yes
Belhadj Kouider et al. (2015)	Yes	Yes	Yes	Yes	Yes	Yes	Yes	Yes	Unclear
Burdzovic Andreas & Brunborg (2017)	Yes	Yes	Yes	Yes	Yes	Yes	Yes	Yes	Yes
Busch et al. (2021)	Yes	Yes	Yes	Yes	Yes	Yes	Yes	Yes	Yes
Ertanir et al. (2021)	Yes	Yes	Yes	Yes	Yes	Yes	Yes	Yes	Yes
Gutmann et al. (2019)	Yes	Unclear	Yes	Yes	Yes	Yes	Yes	Yes	Yes
Hüsler & Werlen (2010)	Yes	Yes	Yes	Yes	Yes	Yes	No	Yes	Yes
Karadag & Ogutlu (2021)	Yes	Yes	Yes	Yes	Yes	Yes	Unclear	Yes	Yes
Miconi et al. (2017)	Yes	Yes	Yes	Yes	Yes	Yes	Yes	Yes	Yes
Paalman et al. (2015)	Yes	Yes	Yes	Yes	Yes	Yes	Yes	Yes	Yes
Romero-Acosta et al. (2014)	Yes	Yes	Yes	Yes	Yes	Yes	Yes	Yes	Yes
Stefanek et al. (2012)	Yes	Yes	Yes	Yes	Yes	Yes	Yes	Yes	Yes
Strohmeier & Dogan (2012)	Yes	Yes	Yes	Yes	Yes	Yes	Yes	Yes	Yes

Thommessen et al. (2013)	Yes	Yes	Yes	Yes	Yes	Yes	Yes	Yes	Yes
Ustuner Top & Yigitbas (2021)	Yes	Yes	Yes	Yes	Yes	Yes	Yes	Yes	Yes
Verhulp et al. (2014)	Yes	Yes	Yes	Yes	Yes	Yes	Yes	Yes	Yes
Verhulp et al. (2015)	Yes	Yes	Yes	Yes	Yes	Yes	Yes	Yes	Yes

APPENDIX D

Matrix for risk of bias assessment within relational problems studies using JBI (The Joanna Briggs Institute, 2011)

**Reference**	**1** Was the sample frame appropriate to address the target population?	**2** Were study participants sampled in an appropriate way?	**3** Was the sample size adequate?	**4** Were the study subjects and the setting described in detail?	**5** Was the data analysis conducted with sufficient coverage of the identified sample?	**6** Were valid methods used for the identification of the condition?	**7** Was the condition measured in a standard, reliable way for all participants?	**8** Was there appropriate statistical analysis?	**9** Was the response rate adequate, and if not, was the low response rate managed appropriately?
Alivernini et al. (2019)	Yes	Yes	Yes	Yes	Yes	Yes	Yes	Yes	Yes
Asendorpf & Motti-Stefanidi (2017)	Yes	Yes	Yes	Yes	Yes	Yes	Yes	Yes	Yes
Bianchi et al. (2021)	Yes	Yes	Yes	Yes	Yes	Yes	Yes	Yes	Yes
Borraccino et al. (2020)	Yes	Yes	Yes	Yes	Yes	Yes	Yes	Yes	Yes
Caravita et al. (2020)	Yes	Yes	Yes	Yes	Yes	Yes	Yes	Yes	Yes
Dalmasso et al. (2018)	Yes	Yes	Yes	Yes	Yes	Yes	Yes	Yes	Yes

Delaruelle et al. (2021)	Yes	Yes	Yes	Yes	Yes	Yes	Yes	Yes	Yes
Duinhof et al. (2020)	Yes	Yes	Yes	Yes	Yes	Yes	Yes	Yes	Yes
Hjern et al. (2013)	Yes	Yes	Yes	Yes	Yes	Yes	Yes	Yes	Yes
Karadag & Gokcen (2021)	Yes	Yes	No	Yes	Yes	Yes	Yes	Yes	Yes
Karadag & Ogutlu (2021)	Yes	Yes	Yes	Yes	Yes	Yes	Unclear	Yes	Yes
Motti-Stefanidi et al. (2012)	Yes	Yes	Yes	Yes	Yes	Yes	Yes	Yes	Yes
Nunes et al. (2016)	Yes	Yes	Yes	Yes	Yes	Yes	Yes	Yes	Yes
Plenty & Jonsson (2017)	Yes	Yes	Yes	Yes	Yes	Yes	Yes	Yes	Yes
Romero-Oliva et al. (2017)	Yes	Yes	Yes	Yes	Yes	Yes	Yes	Yes	Yes
Smith et al. (2015)	Yes	Yes	Yes	Yes	Yes	Yes	Yes	Yes	Yes
Svensson (2012)	Yes	Yes	Yes	Yes	Yes	Yes	Yes	Yes	Yes
Ustuner Top & Yigitbas (2021)	Yes	Yes	Yes	Yes	Yes	Yes	Yes	Yes	Yes

APPENDIX E

Key characteristics of included studies on emotional problems

Study reference	Profile	Sample	Country of destination	Country of origin	Compares by origin	Definition of migrant	Definition of native	Emotional problems outcomes	Measures	Problem behaviour report
(Alonso-Fernández et al., 2017)	Voluntary migration	N=636 non-institutionalised sample of adolescents aged 12-14	Spain	/	/	Those who had a different nationality than the	Those who had the nationality of the	Emotional symptoms	Kidscreen-10 SDQ	Adolescents

		(n natives = 424; n migrants = 212)				host country	host country			
(Belhadj Kouider et al., 2014)	Voluntary migration<br><br>(1st and 2nd generation)	N=5,680 institutionalised sample of children aged 3-18<br>(n natives = 3,962; n migrants = 1,716; 1st gen = 775; 2nd gen = 961)	Germany	African countries<br>Arabic / Oriental countries<br>Asian countries<br>North American countries<br>Other European countries<br>Poland<br>Russia<br>South American countries<br>Turkey (Kurdish and Turkish origin)	Yes	/	/	Affective disorders	ICD-10	Adolescents (diagnosis)
(E. Belhadj Kouider et al., 2015)	Voluntary migration<br><br>(1st and 2nd generation)	N=6,269 institutionalised sample of children aged 3-20<br>(n natives = 4,858; n migrants = 1,438)	Germany	African countries<br>Arabic / Oriental countries<br>Asian countries<br>North American countries<br>Other European countries	Yes		/	Internalizing disorders	ICD-10<br>CBCL	Adolescents (diagnosis)<br>Parents<br>Teachers

				Poland<br>Russia<br>South American countries<br>Turkey (Kurdish and Turkish origin)						
(Burdzovic Andreas & Brunborg, 2017)	Voluntary migration<br><br>(1st and 2nd generation)	N=884 non-institutionalised sample of adolescents aged 13-18 (n natives = 713; n migrants = 171; n 1st gen = 169; n 2nd gen = 4)	Norway	/	/	1st gen migrant: born abroad with both parents born abroad<br><br>2nd gen migrant (“native-born”): born in host country and either one or both parents born abroad	Native child of native parents	Depressive symptomatology<br>Emotional problems	PHQ-9<br>SDQ	Adolescents
(Busch et al., 2021)	Voluntary migration	N=1,839 non-institutionalised sample of children aged 7-18	Germany	Poland<br>Russia<br>Turkey<br>66 other countries	/	/	/	Anxiety<br>Depression	BAI-Y<br>BDI-Y<br>BYI-II	Adolescents

		(n natives = 612; n migrants = 425)								
(Ertanir et al., 2021)	Voluntary migration	N=362 non-institutionalised sample of adolescents (n natives = 199; n migrants = 163)	Switzerland	Albania Bosnia and Herzegovina Germany Italy Kosovo Portugal Turkey Serbia	/	Those who had a different nationality than the host country (they could have both)	/	Anxiety symptoms Depression symptoms	HSCL-25	Adolescents
(Gutmann et al., 2019)	Voluntary migration	N=302 non-institutionalised sample of children aged 7-18 (n natives = 152; n migrants = 150)	Austria	Turkey	/	/	/	Internalizing problems (Anxiety and Depression)	CBCL DIKJ STAI STAI-K YSR	Adolescents
(Hüsler & Werlen, 2010)	Voluntary migration (1st and 2nd generation)	N=1,352 institutionalised sample of adolescents aged 11-20 (n natives = 706; n migrants = 646; 1st gen = 423; 2nd gen = 223)	Switzerland	African countries Balkan countries European countries Near East countries United States	/	/	/	Anxiety Depression	ADS STAI	Adolescents
(Karadag & Ogutlu, 2021)	Refugee adolescents	N=128 non-institutionalise	Turkey	Syria	/	/	/	Anxiety Depression	DASS-42 SDQ	Adolescents

		d sample of adolescents aged 12-16 (n natives = 66; n refugee migrants = 62)						Emotional symptoms<br>Stress		
(Miconi et al., 2017)	Voluntary migration<br><br>(1st generation)	N=1,981 non-institutionalised sample of adolescents aged 14-20 (n natives = 1,295; n 1st gen migrants = 686)	Italy	Albania<br>China<br>India<br>Moldavia<br>Morocco<br>Romania<br>18 other countries	/	1st gen migrants: child and parents born abroad	Native child of native parents	Depression symptoms	Psychological problems scale developed for the ICSEY-study	Adolescents
(Paalman et al., 2015)	Voluntary migration	N=318 non-institutionalised sample of adolescents aged 11-12 (n natives = 159; n migrants = 159)	Netherlands	Morocco	/	1st gen migrant: child and parents born abroad<br><br>2nd gen migrant: child born in host country and at least one of their parents born abroad	Native child of native parents	Depression<br>Generalized anxiety<br>Social Anxiety	RADS-2<br>SCARED	Adolescents

(Romero-Acosta et al., 2014)	Voluntary migration	N=993 non-institutionalised sample of adolescents aged 13-16 (n natives = 834; n migrants = 159)	Spain	Latin American countries	/	/	/	Depression<br>Anxiety (total)<br>· Generalized anxiety<br>· Separation anxiety<br>· Social phobia	CDI<br>SCARED	Adolescents
(Stefanek et al., 2012)	Voluntary migration<br><br>(1st and 2nd generation)	N=609 non-institutionalised sample of adolescents aged 14-19 (n natives = 330; n migrants = 279; n 1st gen = 120; n 2nd gen = 159)	Austria	Albania<br>Croatia<br>Poland<br>Serbia<br>Turkey<br>Others	/	1st gen migrant: child and parents born abroad<br><br>2nd gen migrant: child born in host country and at least one parent born abroad	Native child of native parents	Depression symptoms	YSR	Adolescents
(Strohmeier & Dogan, 2012)	Voluntary migration<br><br>(1st and 2nd generation)	N=663 non-institutionalised sample of adolescents aged 11 and 15 (n natives = 379; n migrants = 284; n 1st gen = 82; n 2nd gen = 202)	Austria	Turkey	/	1st gen migrant: child and parents born abroad<br><br>2nd gen migrant: native-born	Native child of at least one native parent	Depression<br>Social anxiety	CDI	Adolescents

						children and at least one of their parents born abroad				
(Thommessen et al., 2013)	Unaccompanied migrant children	N=120 non-institutionalised sample (only UAMs in reception centres) of adolescents aged 17 to 18 (n natives = 60; n UAMs = 60)	Italy	Afghanistan Bangladesh Guinea-Bissau	/	/	/	Anxiety Affective problems Depression	CBCL	Parents Social workers
(Ustuner Top & Yigitbas, 2021)	Voluntary migration	N=150 non-institutionalised sample of adolescents aged 12-15 (n natives = 75; n migrants = 75)	Turkey	Afghanistan Iraq Syria	/	/	/	Social Anxiety	SAS-A	Adolescents
(Verhulp et al., 2014)	Voluntary migration	N=349 non-institutionalised sample of adolescents aged 13-17 (n natives = 95; n migrants = 253)	Netherlands	Morocco Surinam Turkey	Yes	/	/	Affective disorders Anxiety disorders	The Anxiety Disorders Interview Schedule for Diagnostic and Statistical Manual of Mental Disorders	Adolescents (interview)

									(4th ed.; DSM-IV; American Psychiatric Association)<br><br>Child version DSM-IV	
(Verhulp et al., 2015)	Voluntary migration	N=349 non-institutionalised sample of adolescents aged 13-18 (n natives = 95; n migrants = 253)	Netherlands	Morocco Surinam Turkey	Yes	/	/	Anxiety Depression	YSR	Adolescents Parents

APPENDIX F

Key characteristics of included studies on relational problems

Study reference	Profile	Sample	Country of destination	Country of origin	Compares by origin	Definition of migrant	Definition of native	Relational problems outcomes	Measures	Problem behaviour report
(Alivernini et al., 2019)	Voluntary migration	N=36,712 non-institutionalised sample of adolescents aged 13-19	Italy	/	/	1st gen: child and parents born abroad	Native child with at least one native parent	Peer relatedness · Peer friendship · Peer acceptance	CSIQ-A	Adolescents

		(n natives = 33,348; n migrants = 3,364; 1st gen = 2,239; 2nd gen = 1,395)				2nd gen: native child and both parents born abroad				
(Asendorpf & Motti-Stefanidi, 2017)	Voluntary migration	N=1,057 non-institutionalised sample of adolescents aged 12-15 (n natives = 525; n migrants = 532; 1st gen = 316; 2nd gen = 216)	Greece	Albania<br>Russia (Pontian-Greek origin)<br>Others	/	/	/	Peer acceptance<br>Peer rejection	Peer nominations	Adolescents
(Bianchi et al., 2021)	Voluntary migration	N=249 non-institutionalised sample of adolescents aged 11-18 (n natives = 201; n migrants = 48; 1st gen = 25; 2nd gen = 23)	Italy	/	/	1st gen: child and parents born abroad<br>2nd gen: native child and both parents born abroad	Native child with at least one native parent	Friendship<br>Peer acceptance	CSIQ-A	Adolescents
(Borraccino et al., 2020)	Voluntary migration	N=47,399 non-institutionalised sample of	Italy	Eastern European / non-Western and	Yes	1st gen: child and at least one parent	Native child of native parents	Perceived peer support	Multidimensional scale of	Adolescents

		adolescents aged 11, 13 & 15 (n natives = 40,224; n migrants = 7,175)		non-European countries (EU-13, African countries, Albania, Asian countries, Bosnia, Central American countries, Macedonia, Moldavia, Serbia, South American countries, Ukraine) Western countries (EU-15, Australia, Iceland, New Zealand, Norway, Switzerland, USA)		born abroad<br>2nd gen: native child and at least one parent born abroad			perceived social support	
(Caravita et al., 2020)	Voluntary migration	N=692 non-institutionalised sample of adolescents' mean age 13.07 (n natives = 594; n migrants	Italy	African countries Asian countries Central American countries	/	1st gen: child and parents born abroad	Native child of native parents	Peer status · Peer acceptance · Popularity	Georgia School Climate Survey<br>Peer nominations	Adolescents

		= 98; 1st gen = 45; 2nd gen = 53)		European countries South American countries		2nd gen: native child and both parents born abroad				
(Dalmasso et al., 2018)	Voluntary migration	N=47,399 non-institutionalised sample of adolescents aged 11, 13 & 15 (n natives = 41,048; n migrants = 88,477)	Italy	Eastern European countries Non-Western and non-European countries Western countries	Yes	1st gen: child and at least one parent born abroad<br>2nd gen: native child and at least one parent born abroad	Native child of native parents	Classmate support Peer support	Multidimensional scale of perceived social support	Adolescents
(Delaruelle et al., 2021)	Voluntary migration	N=121,751 non-institutionalised sample of adolescents aged 11, 13 & 15	29 European countries	/	/	1st gen: child and at least one parent born abroad<br>2nd gen: native child and at least one parent born abroad	Native child of native parents<br>Child born abroad with both native parents	Student support	Adapted scales from Torsheim et al. (2000)	Adolescents

(Duinhof et al., 2020)	Voluntary migration	N=6,337 non-institutionalised sample of adolescents aged 11-16 (n natives = 5,283; n migrants = 1,054; 1st gen = 153; 2nd gen = 901)	Netherlands	Morocco<br>Surinam<br>The Antilles<br>Turkey	/	migrant: at least one parent born abroad (includes 1st and 2nd gen)	Native child of native parents	Peer relationship problems	SDQ-R	Adolescents
(Hjern et al., 2013)	Voluntary migration	N=76,229 non-institutionalised sample of adolescents aged 15 (n natives = 59,703; n migrants = 19,356; 1st gen = 4,116; 2nd gen = 15,240)	Sweden	African countries<br>Asian countries<br>Others	Yes	1st gen: child and both parents born abroad<br>2nd gen: native child and one or both parents born abroad	Native child of native parents	Peer relations<br>Social acceptance	Kidscreen-52	Adolescents
(Karadag & Gokcen, 2021)	Voluntary migration	N=171 non-institutionalised sample of adolescents (n natives = 66; n migrants = 105)	Turkey	Syria	/	Migrant: child and parents born abroad	/	Peer problems	SDQ	Adolescents

(Karadag & Ogutlu, 2021)	Refugee adolescents	N=128 non-institutionalised sample of adolescents aged 12-16 (n natives = 66; n migrants = 62)	Turkey	Syria	/	Migrant: child and parents born abroad	/	Peer relationship problems	SDQ	Adolescents
(Motti-Stefanidi et al., 2012)	Voluntary migration	N=1,057 non-institutionalised sample of adolescents aged 12-15 (n natives = 525; n migrants = 532; 1st gen = 316; 2nd gen = 216)	Greece	African countries Albania Asian countries European countries Russia (Pontian Greek origin)	Yes	/	/	Peer popularity	Peer nominations	Adolescents
(Nunes et al., 2016)	Voluntary migration	N=475 non-institutionalised sample of adolescents aged 12-17 (n natives = 272; n migrants = 203)	Portugal Spain	African countries countries Eastern European countries South American Others	/	/	/	Social acceptance Social support from peers	Kidscreen-52	Adolescents
(Plenty & Jonsson, 2017)	Voluntary migration	N=5,021 non-institutionalised sample of adolescents aged 14-15	Sweden	African countries Eastern European countries	Yes	1st gen: child and parents born abroad	Native or adoptive child with at least one native parent	Social exclusion outcomes - Rejection - Isolation	Peer nominations	Adolescents

		(n natives = 3,544; n migrants = 1,477)		Latin American countries<br>Middle Eastern countries<br>Southern European countries<br>Western European or other Western countries		2nd gen: native child and parents born abroad				
(Romero-Oliva et al., 2017)	Voluntary migration	N=1,151 non-institutionalised sample of adolescents aged 12-18<br>(n natives = 678; n migrants = 473)	Portugal<br>Spain	African countries<br>countries<br>Eastern European countries<br>South American<br>Others	/	/	/	Friends<br>Social competence	Kidscreen-52<br>SSRS	Adolescents
(Smith et al., 2015)	Voluntary migration	N=9,054 non-institutionalised sample of adolescents aged 15<br>(n natives = 5,683; n migrants = 3,371)	Germany<br>Netherlands	100 different countries	/	Migrant: at least one parent born abroad (includes 1st and 2nd gen)	Native child of native parents	Friends	Peer nominations	Adolescents
(Svensson et al., 2012)	Voluntary migration	N=1,169 non-institutionalised	Sweden	Eastern European countries	/	1st gen: child and parents	Native child of	Peer friendships / network	Peer nominations	Adolescents

		sample of adolescents aged 12-16 (n natives = 846; n migrants = 323; 1st gen = 150; 2nd gen = 173)		Eritrea<br>Former Yugoslavian countries<br>Middle Eastern countries<br>Somalia<br>Others		born abroad<br>2nd gen: native child and parents born abroad	native parents			
(Ustuner Top & Yigitbas, 2021)	Voluntary migration	N=150 non-institutionalised sample of adolescents aged 12-15 (n natives = 75; n migrants = 75)	Turkey	Afghanistan<br>Iraq<br>Syria	/	/	/	Interpersonal relations	ALP Scale	Adolescents

APPENDIX G

List of validated tools used in the included studies to measure emotional problems

Tool	Studies	Supporting study	Key information
Kidscreen-10	(Alonso-Fernández et al., 2017)	Instituto Nacional de Estadística, INE, 2006, 2011	Both the SDQ and the Kidscreen-10 have been validated for Spanish children and adolescents (Instituto Nacional de Estadística, INE, 2006, 2011).
SDQ	(Alonso-Fernández et al., 2017)<br><br>(Burdzovic Andreas & Brunborg, 2017)<br><br>(Karadag & Ogutlu, 2021)	Instituto Nacional de Estadística, INE, 2006, 2011<br><br>(Heiervang et al., 2008; Goodman et al., 2011; Bøe et al., 2016)<br><br>Güvenir T, Özbek A, Baykara B, Arkar H, Sentürk B, I˙ncekas¸ S. Gücler ve güçlükler anketi’nin (gga) Türkçe uyarlamasinin psikometrik özellikleri. Çocuk ve Gençlik Ruh Sag˘ lig˘ i Dergisi. 2008;15:65-74.<br><br>Alyahri A, Goodman R. Validation of the arabic strengths and difficulties questionnaire and the development and well-being assessment. East Mediterr Health J. 2006;12 Suppl 2:S138-46.	Both the SDQ and the Kidscreen-10 have been validated for Spanish children and adolescents (Instituto Nacional de Estadística, INE, 2006, 2011).<br><br>Strengths and Difficulties Questionnaire (SDQ; Goodman and Goodman, 2009) previously used in Norwegian samples (Heiervang et al., 2008; Goodman et al., 2011; Bøe et al., 2016)<br><br>The Turkish and Arabic versions of the SDQ were also both shown to be valid and reliable questionnaires.
CBCL	(E. Belhadj Kouider et al., 2015)<br><br>(Gutmann et al., 2019)<br><br>(Thommessen et al., 2013)	Schmeck, K., Poustka, F., Döpfner, M., Plück, J., Berner, W., Lehmkuhl, G., ... & Lehmkuhl, U. (2001).<br><br>Discriminant validity of the child behaviour checklist CBCL-4/18 in German samples. *European child & adolescent psychiatry*, *10*, 240-247.	Results indicated that the discriminant validity of the German version of CBCL is comparable to the original English version.<br><br>Erol N, BengiArslan L, Akcakin M. The adaptation and standardization of the child behavioral checklist among 6 to 18yearold Turkish children. In: Sergeant J, editor. Eunethydis: European approaches to hyperkinetic disorder.
PHQ-9	(Burdzovic Andreas &	Brattmyr, M., Lindberg, M. S., Solem, S., Hjemdal, O., & Havnen, A. (2022). Factor structure, measurement invariance, and concurrent validity of the Patient Health	This study shows that the PHQ-9 and GAD-7 may be used as one-dimensional instruments in clinical settings. Tests for measurement

	Brunborg, 2017)	Questionnaire-9 and the Generalized Anxiety Disorder scale-7 in a Norwegian psychiatric outpatient sample. *BMC psychiatry*, *22*(1), 1-11.<br><br>Allgaier, A.-K., Pietsch, K., Frühe, B., Sigl-Glöckner, J., and Schulte-Körne, G. (2012). Screening for depression in adolescents: validity of the Patient Health Questionnaire in pediatric care. Depress. Anxiety 29, 906–913. doi: 10.1002/da.21971<br><br>Ganguly, S., Samanta, M., Roy, P., Chatterjee, S., Kaplan, D. W., and Basu, B. (2013). Patient Health Questionnaire-9 as an effective tool for screening of depression among Indian adolescents. J. Adolesc. Health 52, 546–551. doi: 10.1016/j.jadohealth.2012.09.012<br><br>Richardson, L. P., McCauley, E., Grossman, D. C., McCarty, C. A., Richards, J., Russo, J. E., et al. (2010). Evaluation of the Patient Health Questionnaire (PHQ-9) for detecting major depression among adolescents. Pediatrics 126, 1117–1123. doi: 10.1542/peds.2010-0852<br><br>Tsai, F. J., Huang, Y. H., Liu, H. C., Huang, K. Y., Huang, Y. H., and Liu, S. I. (2014). Patient Health Questionnaire for school-based depression screening among Chinese adolescents. Pediatrics 133, e402–e409. doi: 10.1542/peds.2013-0204	invariance supported that both measures are understood and interpreted comparably across gender and diagnostic subgroups.<br><br>Most importantly, our adolescent PHQ-9 self-reports were not validated against the external diagnostic criteria, such as the official psychiatric diagnoses for example. However, it should be noted that the PHQ-9 has been internationally validated against various diagnostic interviews in multiple adolescent studies (Richardson et al., 2010; Allgaier et al., 2012; Ganguly et al., 2013; Tsai et al., 2014)
BAI-Y<br>BDI-Y<br>BYI-II	(Busch et al., 2021)	Osman, A., Hoffman, J., Barrios, F. X., Kopper, B. A., Breitenstein, J. L., & Hahn, S. K. (2002). Factor structure, reliability, and validity of the Beck Anxiety Inventory in adolescent psychiatric inpatients. *Journal of clinical psychology*, *58*(4), 443-456.<br><br>Osman, A., Kopper, B. A., Barrios, F., Gutierrez, P. M., & Bagge, C. L. (2004). Reliability and validity of the Beck depression inventory--II with adolescent psychiatric inpatients. *Psychological assessment*, *16*(2), 120.	Evidence for convergent and discriminant validity of the BAI was investigated separately in the male and female inpatient samples. Overall, the BAI showed acceptable psychometric properties in these populations.<br><br>In Study 1, expert raters (N=7) and adolescent psychiatric inpatients (N=13) evaluated the BDI-II items to assess content validity. In Study 2, confirmatory factor analyses of several first-order solutions failed to provide adequate fit estimates to data for 205 boys, 203 girls, and the combined sample. Exploratory factor analyses identified new item-factor solutions. Reliability estimates were good (range = .72 to .91) for the BDI-II total and scale scores. In Study 3 (N=161 boys and 158 girls from

		Asikhia, O. A., & Mohangi, K. (2015). A case study of school support and the psychological, emotional and behavioural consequences of HIV and AIDS on adolescents. *SAHARA: Journal of Social Aspects of HIV/AIDS Research Alliance*, *12*(1), 123-133.	Study 2), preliminary evidence for estimates of concurrent, convergent, and discriminant validity were established for the BDI-II.<br><br>The Beck Youth Inventories (BYI-II) […] It has internal consistency of Cronbach's alpha coefficient that ranged from .91 to .96 for ages 15–18 across all five scales and a convergent validity measure of .72.
HSCL-25	(Ertanir et al., 2021)	Glaesmer, H., Braehler, E., Grande, G., Hinz, A., Petermann, F., & Romppel, M. (2014). The German Version of the Hopkins Symptoms Checklist-25 (HSCL-25)—Factorial structure, psychometric properties, and population-based norms. *Comprehensive psychiatry*, *55*(2), 396-403.	On the other hand, the HSCL-25 is a well-established instrument in the field of research about mental health problems in refugees etc. and a huge number of translations are available. The psychometric properties of the HSCL-25 are satisfactory and thus it seems supportable to use the HSCL-25 in this specific field.
DIKJ	(Gutmann et al., 2019)	https://dorsch.hogrefe.com/stichwort/depressionsinventar-fuer-kinder-und-jugendliche-dikj	Reliabilität: Innere Konsistenz: α = ,87/,92. Validität: konvergente (Selbstwert) und divergente (Fähigkeitsselbstkonzept, Motivationale Orientierungen) Zusammenhänge geprüft. Trennt zw. depressiven Pat. und Kontrollen. Normierung an N = 3.395 Kindern (8–16 Jahre). Bearbeitungsdauer: 10–15 Min.<br><br>https://dorsch.hogrefe.com/stichwort/depressionsinventar-fuer-kinder-und-jugendliche-dikj
STAI<br>STAI-K	(Gutmann et al., 2019)<br>(Hüsler & Werlen, 2010)	Oei, T. P., Evans, L., & Crook, G. M. (1990). Utility and validity of the STAI with anxiety disorder patients. *British Journal of Clinical Psychology*, *29*(4), 429-432.<br><br>Spielberger, C. D., Gorsuch, L., Laux, L., Glanzmann, P., & Schaffner, P. (2001). *Das State-Trait-Angstinventar: STAI*. Göttingen, Germany: Beltz Test.	This study demonstrates that the STAI is a useful and valid instrument for measuring anxiety disorder patients and also provides support for the theoretical constructs of state and trait anxiety.
YSR	(Gutmann et al., 2019)<br><br>(Stefanek et al., 2012)<br><br>(Verhulp et al., 2015)	Gomez, R., Vance, A., & Gomez, R. M. (2014). Analysis of the convergent and discriminant validity of the CBCL, TRF, and YSR in a clinic-referred sample. *Journal of abnormal child psychology, 42*, 1413-1425.<br><br>Ferdinand, R. F. (2008). Validity of the CBCL/YSR DSM-IV scales anxiety problems and affective problems. *Journal of anxiety disorders, 22*(1), 126-134.	Arbeitsgruppe Deutsche Child Behavior Checklist. Fragebogen für Jugendliche; deutsche Bearbeitung der Youth SelfReport Form der Child Behavior Checklist (YSR). Einführung und Anleitung zur Handauswertung mit deutschen Normen, bearbeitet von M. Döpfner, J. Plück, S. Bölte, K. Lenz, P. Melchers & K. Heim (2. Aufl.). Köln: Arbeitsgruppe Kinder, Jugend und Familiendiagnostik; 1998.<br><br>The results (significant consistency coefficients) in the study indicated support for the convergent validity of the CBCL and TRF, and CBCL and YSR for all eight scales.

			CBCL and YSR scores on the Affective Problems scale corresponded closely to DSM-IV major depressive disorder and dysthymia. The CBCL/YSR DSM-IV scale Affective Problems showed very strong convergent validity with DSM-IV diagnoses of major depressive disorder and dysthymia.
ADS = CES-D20	(Hüsler & Werlen, 2010)	Maksimović, S., Ziegenbein, M., Machleidt, W., & Sieberer, M. (2014). Messäquivalenz der Allgemeinen Depressionsskala (ADS 20) bei Menschen mit und ohne Migrationshintergrund unter geschlechtsspezifischer Perspektive. *Psychiatrische Praxis*, *41*(06), 324-330.	Mit 2786 Datensätzen und der multigruppenkonfirmatorischen Faktorenanalyse wurden die starke faktorielle Invarianz der ADS 20 überprüft und latente Faktormittelwertdifferenzen geschätzt.<br>Die ADS 20 erwies sich, außer bei den männlichen Migranten, als messäquivalent. Die Migrantinnen hatten in 3 Subskalen signifikant erhöhte latente Faktormittelwerte
DASS-42	(Karadag & Ogutlu, 2021)	Moussa MT, Lovibond P, Laube R, Megahead HA. Psychometric properties of an arabic version of the depression anxiety stress scales (DASS). Res Soc Work Pract. 2017;27:375-86.<br>Bilgel N, Bayram N. Turkish version of the depression anxiety stress scale (DASS-42): psychometric properties. Noro Psikiyatr Ars. 2010;47:118-26.	The DASS-42 questionnaire, which has validity and reliability in both Turkish and Arab populations,12,13 was used to compare depression, anxiety, and stress levels of adolescents. DASS-42
Psychological problems scale developed for the ICSEY-study	(Miconi et al., 2017)	Milfont and Fischer 2010; Van de Schoot et al. 2012	Items have been used with immigrant youth in 13 countries and are answered on a five-point scale ranging from “never” to “very often” (e.g., “I feel unhappy and sad”). A higher score was indicative of more depressive symptoms. The Cronbach’s alphas were .74 for the immigrant sample and .75 for the non-immigrants sample. We translated the measure in the Italian language following a translation back-translation procedure (Van de Vijver and Leung 1997). In addition, since this measure had not been previously validated in the Italian context, a multi-group confirmatory factor analysis using a diagonally weighted least squares method based on polychoric correlations was conducted to establish the measurement invariance of the scale across non-immigrant and immigrant adolescents (Milfont and Fischer 2010; Van de Schoot et al. 2012).
RADS-2	(Paalman et al., 2015)	Osman, Gutierrez, Bagge, Fang, & Emmerich, 2010	Good validity and reliability have been reported in various international studies (Osman, Gutierrez, Bagge, Fang, & Emmerich, 2010).

SCARED	(Paalman et al., 2015) (Romero-Acosta et al., 2014)	Monga, S., Birmaher, B., Chiappetta, L., Brent, D., Kaufman, J., Bridge, J., & Cully, M. (2000). Screen for child anxiety-related emotional disorders (SCARED): Convergent and divergent validity. *Depression and anxiety*, *12*(2), 85-91.<br><br>Canals, J., Hernández-Martínez, C., Cosi, S., & Domènech, E. (2012). Examination of a cutoff score for the Screen for Child Anxiety Related Emotional Disorders (SCARED) in a non-clinical Spanish population. *Journal of Anxiety Disorders*, *26*(8), 785-791.	The SCARED is a reliable and valid screening tool for clinically referred children and adolescents with anxiety disorders.
CDI	(Romero-Acosta et al., 2014) (Strohmeier & Dogan, 2012)	Carey, M. P., Faulstich, M. E., Gresham, F. M., Ruggiero, L., & Enyart, P. (1987). Children's Depression Inventory: construct and discriminant validity across clinical and nonreferred (control) populations. *Journal of Consulting and Clinical Psychology*, *55*(5), 755.<br><br>Öy, B. 1991. Cocuklar icin depresyon ölcegi: Gecerlik ve güvenirlik calismasi. Türk Psikiyatri Dergisi 2: 132–37.<br><br>Stiensmeier-Pelster, J., M. Schürmann, and K. Duda. 2000. DIKJ. Depressions-Inventar für Kinder und Jugendliche. Göttingen: Hogrefe Verlag für Psychologie.	Children’s Depression Inventory (CDI) is one of the most widely used self-report questionnaires for depressive symptoms for 7- to 17-year-old children and adolescents. It takes 10–20 minutes to be completed and consists of 27 items scored on a 3-point Likert scale (0: absent; 1: moderate; 2: severe). A score of 17 or more is considered as having the best sensitivity (92.6%) and specificity (63.3%) in the Spanish general population (Canals et al., 1991). CDI has good internal consistency and good test–retest reliability (Canals et al., 1991; Kovacs, 1992). In our sample, internal consistency was also satisfactory (Cronbach’s alpha value of .83).<br><br>The construct and discriminant validity of the GDI was investigated, using principal component and discriminant analyses, in a large sample of inpatient psychiatric/residential subjects and non-referred subjects. The data from this project indicated that the two-factor and three-factor models provided the optimal factor solutions for combined, clinical, and non-referred samples, with few items loading on more than a single factor. In contrast to Kovacs (1985), the results from this project indicated that the factor structure of the GDI remained generally stable across maximally different (i.e., psychiatric inpatients and non-referred subjects) and similar populations (i.e, combined). Moreover, this study indicated that clinical and non-referred subjects could be reliably discriminated using GDI factor scores.<br><br>The CDI has previously been applied and validated in both the Turkish (Öy 1991) and German languages (Stiensmeier-Pelster, Schürmann, and Duda 2000).

SAS-A	(Ustuner Top & Yigitbas, 2021)	Aydın, A., & Sutcu Tekinsav, S. (2007). Validity and reliability of social anxiety scale for adolescents (SAS-A). JournalofChild and Adolescent Mental Health, 14(2), 79–89.	The scale was developed by La Greca, and Lopez (La Greca and Lopez 1998) and the validity and reliability analyses for Turkish children were developed in 2007.

APPENDIX H

List of validated tools used in the included studies to measure relational problems

Tool	Studies	Supporting study	Key information
CSIQ-A	(Alivernini et al., 2019)<br><br>(Bianchi et al., 2021)	Alivernini, F., & Manganelli, S. (2016). The classmates social isolation questionnaire (CSIQ): An initial validation. European Journal of Developmental Psychology, 13, 264–274. http://doi.org/10.1080/17405629.2016.1152174<br><br>Cavicchiolo, E., Girelli, L., Lucidi, F., Manganelli, S., & Alivernini, F. (2019). The Classmates Social Isolation Questionnaire for Adolescents (CSIQ-A): Validation and invariance across immigrant background, gender and socioeconomic level. Journal of Educational, Cultural and Psychological Studies, 19, 1–15. https://doi.org/10. 7358/ecps-2019-019-cavi.	The CSIQ-A proved to be a psychometrically sound measure and, in this study, the fit of the posited CSIQ-A's measurement model (peer acceptance and peer friendship as two correlated factors) was good.<br><br>The CSIQ-A showed full measurement invariance (Cheung & Rensvold, 2002) across gender and across students with different immigrant backgrounds and socioeconomic status (gender: ΔCFI=0.014; immigrant background: ΔCFI=0.003; SES: ΔCFI=0.003).
Multidimensional scale of perceived social support<br><br>Zimet et al. (1990)	(Borraccino et al., 2020)<br><br>(Dalmasso et al., 2018)	Zimet GD, Dahlem NW, Zimet SG, Farley GK (1988) The multidimensional scale of perceived social support. J Pers Assess 52:30–41<br><br>Zimet GD, Powell SS, Farley GK et al (1990) Psychometric characteristics of the	The multidimensional scales of perceived family and peer support have shown good validity and reliability (Zimet et al. 1988, 1990).

		multidimensional scale of perceived social support. J Pers Assess 55:610–617<br>Publisher's<br><br>Canty-Mitchell J, Zimet GD. Psychometric properties of the Multidimensional Scale of Perceived Social Support in urban adolescents. Am J Commun Psychol. 2000;28(3):391–400	
Adapted scales from Torsheim et al. (2000)	(Delaruelle et al., 2021)	Inchley, J., Currie, D., Cosma, A., & Samdal, O. (2018). Health Behaviour in School-aged Children (HBSC) study protocol: background, methodology and mandatory items for the 2017/18 survey. St Andrews: CAHRU.	Teacher support and student support were assessed using adapted scales from Torsheim and colleagues (2000), which have been validated by the HBSC international network (Inchley et al., 2018).
SDQ<br>SDQ-R	(Karadag & Gokcen, 2021)<br>(Karadag & Ogutlu, 2021)<br>(Duinhof et al., 2020)	Güvenir T, Özbek A, Baykara B, Arkar H, Sentürk B, I˙ncekas¸ S. Güçler ve güçlükler anketi'nin (gga) Türkc¸e uyarlamasinin psikometrik ö zellikleri. C¸ ocuk ve Gençlik Ruh Sag˘ lig˘ i Dergisi. 2008;15:65-74.<br><br>Alyahri A, Goodman R. Validation of the Arabic strengths and difficulties questionnaire and the development and well-being assessment. East Mediterr Health J. 2006;12 Suppl 2:S138-46.<br><br>Duinhof, E. L., Lek, K. M., De Looze, M. E., Cosma, A., Mazur, J., Gobina, I., ... & Stevens, G. W. J. M. (2020). Revising the self-report strengths and difficulties questionnaire for cross-country comparisons of adolescent mental health problems: the SDQ-R. *Epidemiology and psychiatric sciences*, *29*, e35.	The Turkish and Arabic versions of the SDQ were also both shown to be valid and reliable questionnaires.<br><br>Using this revised version of the self-report SDQ, the SDQ-R, partial measurement invariance was established, indicating that latent factor means assessing conduct problems, emotional symptoms, peer relationships problems and hyperactivity-inattention problems could be validly compared across the countries in this study.<br>The SDQ-R was found to have a sufficient amount of invariant items, indicating that adolescent mental health problems could be validly compared across the seven countries in this study. By establishing the SDQ-R, this study contributes to the scarce literature on the cross-cultural validity of scales that examine adolescent mental health problems (Stevanovic et al., 2017).
Kidscreen-52	(Hjern et al., 2013)<br>(Nunes et al., 2016)	Ravens-Sieberer U, Gosch A, Rajmil L, et al. The Quality of Life Measure for children and adolescents: psychometric results from a cross-cultural survey in 13 European countries. Value Health 2008;11:645–58.	The KIDSCREEN questionnaire has been shown to have acceptable levels of reliability and validity.<br><br>Both the SDQ and the Kidscreen-10 have been validated for Spanish children and adolescents (Instituto Nacional de Estadística, INE, 2006, 2011).

	(Romero-Oliva et al., 2017)	16<br><br>Ravens-Sieberer U, Erhart M, Rajmil L, et al. Reliability, construct and criterion validity of the KIDSCREEN-10 score: a short measure for children and adolescents' well-being and health-related quality of life. Qual Life Res 2010;19: 1487–500.<br><br>Instituto Nacional de Estadística, INE, 2006, 2011	
SSRS	(Romero-Oliva et al., 2017)	Posner K, Brown GK, Stanley B, et.al. The Columbia–Suicide Severity Rating Scale: initial validity and internal consistency findings from three multisite studies with adolescents and adults. Am J Psychiatry. 2011; 168:1266–1277.<br><br>Mundt JC, Greist JH, Gelenberg AJ, et al. Feasibility and validation of a computer-automated Columbia–Suicide Severity Rating Scale using interactive voice response technology. J Psychiatr Res. 2010;44(16):1224–1228.	The C-SSRS demonstrated good convergent and divergent validity with other multi-informant suicidal ideation and behavior scales and had high sensitivity and specificity for suicidal behavior classifications compared with another behavior scale and an independent suicide evaluation board.<br><br>They support the feasibility and validity of the eC-SSRS for prospective monitoring of suicidality for use in clinical trials or clinical care
ALP Scale	(Ustuner Top & Yigitbas, 2021)	Ardic, A., & Esin, M. N. (2015). The Adolescent Lifestyle Profile scale: reliability and validity of the Turkish version of the instrument. *Journal of Nursing Research*, *23*(1), 33-40.	The scale was created by Pender to evaluate the health-promoting behavior of adolescents, and the Turkish validity and reliability analyses were developed in 2008.<br><br>Content validity was confirmed by a satisfactory level of agreement with a content validity index of .91. Varimax rotation yielded seven factors with eigenvalues greater than 1, which explained 46.87% of the total variance. The results confirm that the Turkish ALP scale has acceptable psychometric properties and that the scale may be used with Turkish adolescents as an effective measure of their health-promoting lifestyle behaviors.
